# A genome‐scale CRISPR knock‐out screen in chronic myeloid leukemia identifies novel drug resistance mechanisms along with intrinsic apoptosis and MAPK signaling

**DOI:** 10.1002/cam4.3231

**Published:** 2020-07-28

**Authors:** Matthieu Lewis, Valérie Prouzet‐Mauléon, Florence Lichou, Elodie Richard, Richard Iggo, Béatrice Turcq, François‐Xavier Mahon

**Affiliations:** ^1^ Laboratory of Mammary and Leukemic Oncogenesis Inserm U1218 ACTION University of Bordeaux Bergonié Cancer Institute Bordeaux France

**Keywords:** apoptosis, chronic myeloid leukemia, CRISPR/Cas9, screening, TKI resistance

## Abstract

Understanding resistance mechanisms in cancer is of utmost importance for the discovery of novel “druggable” targets. Efficient genetic screening, now even more possible with CRISPR‐Cas9 gene‐editing technology, next‐generation sequencing and bioinformatics, is an important tool for deciphering novel cellular processes, such as resistance to treatment in cancer. Imatinib specifically eliminates chronic myeloid leukemia (CML) cells by targeting and blocking the kinase activity of BCR‐ABL1; however, resistance to treatment exists. In order to discover BCR‐ABL1 independent mechanisms of imatinib resistance, we utilized the genome‐scale CRISPR knock‐out library to screen for imatinib‐sensitizing genes in vitro on K562 cells. We revealed genes that seem essential for imatinib‐induced cell death, such as proapoptotic genes (BIM, BAX) or MAPK inhibitor SPRED2. Specifically, reestablishing apoptosis in BIM knock‐out (KO) cells with BH3 mimetics, or inhibiting MAPK signaling in SPRED2 KO cells with MEK inhibitors restores sensitivity to imatinib. In this work, we discovered previously identified pathways and novel pathways that modulate response to imatinib in CML cell lines, such as the implication of the Mediator complex, mRNA processing and protein ubiquitinylation. Targeting these specific genetic lesions with combinational therapy can overcome resistance phenotypes and paves the road for the use of precision oncology.

## Graphical abstract

We utilized a genome‐scale CRISPR library to discover genes and pathways that could potentially be the target of novel therapeutic strategies for overcoming resistance to imatinib in chronic myeloid leukemia in vitro.

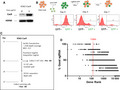

## 1 INTRODUCTION

Efficient genetic engineering in living mammalian cells has become increasingly accessible with the discovery and the improved understanding of the CRISPR (clustered regularly interspaced short palindromic repeats)‐Cas9 system.^1^ This system includes two components, an endonuclease (Cas9) that performs double stranded breaks in DNA if bound to a single guide (sg)RNA molecule that guides the Cas9 protein to specific 20‐nucleotide stretches. These sgRNAs can be designed in silico to target DNA sequences upstream of protospacer adjacent motif (PAM) nucleotides “NGG” for *Streptococcus pyogenes* Cas9 (SpCas9). The CRISPR complexes can efficiently perform irreversible gene knock‐outs, which have revolutionized the study of multiple pathologies and cellular processes in living mammalian cells.^2^

The flexibility of this highly effective tool has been harnessed by laboratories worldwide to design libraries of sgRNAs that can target different elements of the genome, such as protein‐coding genes, miRNAs, or promoter regions.^3^ As a consequence of the decreasing cost of next‐generation sequencing, the availability of numerous different CRISPR libraries and free‐to‐use bioinformatic programs, CRISPR screening is becoming more and more prevalent.^4^ Multiple teams have already used such genome‐scale CRISPR screening approaches to discover, for example, essential genes in cancer cell lines, regulatory pathways involved in a multitude of cellular processes and genes involved in drug resistance.^3^, ^5^, ^6^, ^7^, ^8^ We focused our work on better understanding the response to treatment in chronic myeloid leukemia (CML).

CML is a myeloproliferative disorder which is identified by the increased and uncontrolled proliferation of granulocytes (neutrophils, basophils, and eosinophils) and myeloid precursors in the bone marrow and blood.^9^ This type of neoplasm is most commonly characterized by the presence of a Philadelphia chromosome, caused by the genetic translocation t(9;22)(q34;q11). This genetic abnormality juxtaposes two genes (*BCR* and *ABL1*), whose fusion codes for the constitutively active tyrosine kinase BCR‐ABL1.^10^ Targeting this protein with tyrosine kinase inhibitors (TKI) such as imatinib mesylate revolutionized the treatment of this disorder.^11^ Most patients under treatment have a comparably normal life span,^12^ however, like for all types of targeted therapies in cancer, resistance to treatment can occur.^13^ Indeed, approximately 20% of CML patients present resistance or experience relapse to imatinib treatment. Mutations in the kinase domain, overexpression or amplification of the *BCR‐ABL1* gene account for half of all imatinib‐resistant types.^14^, ^15^ These patients can be, for the most part, treated with increased doses of imatinib or second‐generation TKI such as dasatinib or nilotinib.^14^

We focused our work on further understanding the alternative form of imatinib resistance, BCR‐ABL1 independent resistance, which accounts for the other half of all resistance types.^16^ For such patients, unknown alternative mutations elsewhere in the genome are hypothesized to be responsible for the nonoptimal response to imatinib treatment. Equally, the accumulation of alternative mutations in CML cells may cause the progression of the pathology from a chronic phase to an accelerated phase and eventually a fatal blast crisis.^17^ Therefore, discovering alternative “druggable” targets is crucial in attempting to propose efficient drug combinations for patients with poor prognosis.

In this study, we used the genome‐scale CRISPR knock‐out (GeCKO v2) library,^3^ containing 121 413 unique sgRNAs targeting 20 914 protein coding genes and miRNAs (combination of GeCKO v2 sub‐banks A and B), to screen for imatinib resistance in vitro on K562 cells, a BCR‐ABL1^+^ imatinib‐sensitive cell line for studying CML. We utilized bioinformatic tools^18^ to unveil actors in imatinib‐induced cell death and validated the top hits by individual knock‐out and in vitro characterization. We then targeted two of the identified resistance pathways, intrinsic apoptosis and MAPK signaling, to test for effective drug combinations on imatinib‐resistant cells. We prove that CRISPR screens can be used to discover novel pathways involved in drug resistance and enhance current knowledge concerning cancer cell pathophysiology and susceptibility of response to targeted treatment.

## 2 MATERIALS AND METHODS

### 2.1 Reagents

Imatinib mesylate (imatinib [Cat no. S1026], Euromedex), ABT‐263 (navitoclax [Cat no. S1001], Abbot), and MEK inhibitor (pimasertib [Cat no. S1475], Merck Serono) were purchased from Selleck Chemicals LLC. Puromycin (Cat no. 7130), blasticidin (Cat no. 15202) and doxycycline (Cat no. 9891) were purchased from Sigma‐Aldrich. Annexin V‐APC (Cat no. 640941) and Propidium Iodide (Cat no. 421301) were purchased from BioLegend (Ozyme).

### 2.2 Doxycycline‐inducible spCas9 clonal cell lines

A vector containing SpCas9 (3xFLAG) under the Tet‐On promoter (pCW‐Cas9) was obtained from Addgene (#50661)^5^ and engineered in order to replace puromycin resistance with blasticidin resistance. Amplification and lentiviral production were accomplished and cells were transduced at a multiplicity of infection (MOI) = 1. Cells were treated at 10 mg/mL blasticidin for 7 days. Cells were subsequently plated in methylcellulose (Stemcell) at a density of 200 cells/mL. Individual clones were hand‐picked after 10 days and plated in 96 well plates with supplemented RPMI medium. After 7 more days, amplified cells were transferred into individual flasks and a subset of each clone underwent doxycycline induction for 72 hours. Cas9 expression was detected by western blot and Cas9 activity was observed using a GFP/sgRNA‐anti‐GFP cleavage assay.^3^ Briefly, doxycycline‐inducible Cas9 clonal cell populations were transduced by lenti‐GFP and selected with G418 (750 µg/mL) for 5 days. GFP+ cells were subsequently transduced by lenti‐sgRNA‐anti‐GFP (protospacer for sgRNA anti‐GFP: *GGCCACAAGTTCAGCGTGTC*) and GFP expression was monitored by flow cytometry on a FACS Canto II (Becton Dickinson). Cells that showed correct Cas9 expression and activity were frozen and used for the GeCKO screens.

### 2.3 Genome‐scale CRISPR knock‐out (GeCKO) library

The human GeCKO v2 two‐plasmid system library was purchased from Addgene (#52962).^3^ This two‐plasmid system contains a lentiCas9‐blast plasmid, which we did not use, and 122 417 different lentiGuide‐puro plasmids, each containing a sgRNA. The amplification of these plasmids and lentiviral production was performed following the protocol described by Shalem et al.^3^

### 2.4 Cell culture

CML model cell lines K562 (ATCC® CCL‐243) and LAMA‐84 (ATCC® CRL‐3347) were acquired from DSMZ and used at a low passage. Cells were cultivated in RPMI 1640 medium (ThermoFisher), 10% heat‐inactivated fetal bovine serum (FBS) (Eurobio, Cat No. S182H), 100 IU/mL penicillin, and 100 µg/mL streptomycin in a humidified 5% CO_2_ atmosphere at 37°C. Cells were passaged every 2‐3 days in order to maintain logarithmic growth between 2 × 10^5^ and 1 × 10^6^ cells/mL.

### 2.5 CRISPR‐Cas9 screening procedure

We then transduced the previously selected K562 Cas9^+^ cell line with the GeCKO v2 library. The MOI used for the CRISPR library transduction was of 0.3 to limit multiple lentiviral integrations, thus sgRNAs, into the same cell. At the time of infection, each sgRNA was represented ≈250X, which is the minimum recommended coverage. Cells were maintained for 10 days in medium containing puromycin (5 µg/mL), to select for sgRNA containing cells, and doxycycline (1 µg/mL), to induce Cas9 expression. This timeframe allows for the knock‐out of all of the genes targeted by the GeCKO v2 library sgRNAs. At this time point, 3 × 10^7^ cells were collected (Pre‐IM) and 12 × 10^7^ cells were incubated with 1 µmol/L imatinib for 4 days. After imatinib challenge, surviving cells were isolated by density gradient centrifugation on FICOLL®, washed thoroughly, plated without imatinib at 2 × 10^5^ cells/mL for 3 days, and finally collected (Post‐IM).

### 2.6 sgRNA amplification and next‐generation sequencing (NGS)

DNA from both pre‐ and post‐IM populations were extracted using Genomic 500/G tips (Qiagen, Cat No. 10262). In order to amplify all sgRNAs, 12 PCR reactions were performed for each sample using 12 × 10 µg of extracted DNA. Primers used for sgRNA amplification are the same as those from Shalem et al.^3^ Amplicons were gel extracted, purified, and sequenced on a MiSeq (Illumina). Fastq files generated from NGS were trimmed using CutAdapt^19^ and enriched sgRNAs in the resistant population (Post‐IM) were ranked using the MAGeCK script.^18^ Briefly, MAGeCK ranks each sgRNA based on *P* values calculated from a negative binomial model, then uses a modified robust ranking aggregation algorithm to identify positively selected genes. These genes are equally classified according to their false discovery rate (FDR), which gives a method of conceptualizing the rate of type I errors in null hypothesis testing when conducting multiple comparisons.

### 2.7 Individual sgRNA cloning

Individual gene knock‐out validation was performed by cloning the top sgRNA from the eight top genes of the GeCKO screening procedure into lentiGuide‐Puro backbones from Addgene (#52963).^3^ For the dual CRISPR SPRED2 knock‐out, sgRNAs were designed using ChopChopV2 software.^20^ Briefly, forward and reverse single stranded sgRNA primers (Table S1) were purchased from Eurogentec, annealed, and phosphorylated on their 5′ ends by T4 Polynucleotide Kinase (New England Biolabs, Cat No. M0201S). The double stranded product possesses 5′ overhangs that permit the correct ligation into *Bsm*BI (New England Biolabs, Cat No. R0580S) digested lentiGuide‐Puro vectors. Ligation was performed using Quick Ligase (New England Biolabs, Cat No. E6047). Plasmids were subsequently amplified into TOP10® *Escherichia coli* and protospacer integration was verified by PCR using the reverse single stranded sgRNA primer and a universal U6 promoter primer. Positive colonies were transferred into 50 mL of lysogeny broth (LB) and incubated at 37°C overnight. Plasmids were extracted using Nucleobond Xtra Midi EF (Macherey‐Nagel, Cat No. 740420.50) columns and packaged into lentiviruses.

K562 Cas9^+^ cells or LAMA‐84 Cas9^+^ cells were transduced by lenti‐sgRNA viruses. Multiplicity of infection of the top hit verification was 0.3 (same MOI as screening procedure). Multiplicity of infection of the efficient knock‐outs for *BIM*, *BAX,* and *SPRED2* was 1. Cells were incubated for 72 hours at 5 µg/mL puromycin and 1 µg/mL doxycycline, then solely with doxycycline for 10 additional days.

### 2.8 Viral production procedure

Twenty‐four hours prior to transfection, 4.5 × 10^6^ HEK 293T cells (ATCC® CRL‐3216™) were seeded on a 10‐cm dish. One hour prior to transfection, cell media was changed to 5 mL DMEM 10% FBS containing 25 µmol/L chloroquine (Sigma, Cat no. C6628). The following vector transfection mix was prepared in a 1.5‐mL eppendorf tube: (4 µg VSV‐G: viral envelope, Addgene #14888), 10 µg pSD16 (packaging construct, Addgene #14887) and 10 µg of viral vector was added to 450 µL H_2_O. 500 µL HeBS (Sigma, Cat no. 51558) and 50 µL CaCl_2_ 2.5 mol/L (Sigma, Cat No. C5080) were added drop by drop to the transfection mix, which is then homogenously diluted, drop by drop, in the plate containing 293T cells. Cells were incubated at 37°C for 6 hours, then culture medium was replaced with 8 mL of fresh OptiMEM (Thermo, Cat no. 31985062) 20 mmol/L HEPES (Sigma, Cat no. H3784) medium. Twenty‐four to 40 hours later, the total culture medium was removed, centrifuged at 2000 *g* for 3 minutes, and filtered with 0.22‐µm syringe filter. One‐milliliter vials were stored at −80°C.

### 2.9 CRISPR cut validation

A lysate of CRISPR modified cells was obtained using Phire Tissue Direct PCR Master Mix kit (Thermofisher) and directly used for subsequent PCR reaction. CRISPR cut sites were amplified using primers that flank each cut site. Amplicons were Sanger sequenced (GATC) and analyzed using FinchTV software. CRISPR cut efficiency was calculated using TIDE: tracking of indels by decomposition software (Desktop Genetics).^21^

### 2.10 Gene set enrichment and functional protein network analyses

Gene set enrichment analysis was performed on the GenePattern platform from the Broad Institute, using the GSEAPreranked module.^22^ The user‐supplied ranked list of genes was the MAGeCK rank generated by the imatinib resistance screen. Functional protein network analysis was performed on STRING‐db from the Swiss Institute of Bioinformatics (https://string‐db.org/). The input was the list of the top 158 genes and disconnected nodes were hidden from the network.

### 2.11 Cell proliferation assay

Cells were plated into 96 well plates at 1 × 10^4^ cells per well (100 µL). Cells were incubated in fresh medium or medium containing 0.5 µmol/L imatinib for 96 hours. At each time point, 20 µL of CellTiter 96® AQueous One Solution Cell Proliferation Assay (Promega) was added to the cell culture and incubated at 37°C for 3 hours. Absorbance was recorded at 490 nm with an iMark Microplate reader (BioRad).

### 2.12 Characterization of apoptosis

Cells were incubated in 24 well plates with different drug combinations, either in medium only, or medium containing 0.5 µmol/L imatinib, 1 µmol/L navitoclax, 10 µmol/L pimasertib, or combinations. LAMA‐84 cells were incubated for 24 hours and K562 cells were incubated for 48 hours. Cells were spun‐down and medium was replaced by annexin binding buffer (HEPES 0.01 mol/L, NaCl 0.14 mol/L, pH 7.4) containing Annexin V‐APC (BioLegend) at 5 µL/mL and propidium iodide (Biolegend) at 50 µg/mL. Cells were incubated for 15 minutes at room temperature and analyzed on a Accuri C6 plus cytometer (BD Biosciences). Induced apoptosis was calculated utilizing the following formula:

$$\text{Induced apoptosis}\left(\%\right)=\frac{\left(\left(\%\;\text{apoptosis treated cells}-\%\;\text{apoptosis untreated cells}\right)\times 100\right)}{\left(100-\%\;\text{apoptosis untreated cells}\right)}.$$

### 2.13 Western blot

5 × 10^5^ cells were collected, lysed using RIPA Cell lysis buffer (Thermo, Cat no. 89900), dosed using Pierce™ BCA Protein Assay kit (Thermo, Cat no. 23225), and diluted in 5X SDS‐page loading buffer (10% SDS, 500 mmol/L DTT, 50% Glycerol, 250 mmol/L Tris‐HCL and 0.5% bromophenol blue dye, pH = 6.8) at 1 µg/µL. 20 µg of protein extract was loaded onto a 10% SDS‐page gel, and migration was performed at 100 V for 90 minutes. After SDS‐PAGE electrophoresis, proteins were transferred onto a PVDF membrane (Biorad). Membranes were saturated with 5% (w/v) fat‐free dry milk, then probed with primary antibodies: mouse anti‐Cas9 (EpiGentek, Cat no. A‐9000), rabbit anti‐HSP60 (Santa Cruz, Cat no. SC13115), rabbit anti‐BIM (Sigma‐Aldrich, Cat no. B7929). All antibodies were used at a 1/1000 dilution. After secondary antibody labeling, peroxidase activity was revealed using Western Lightning Plus‐ECL kit (Perkin Elmer, Cat no. NEL103001EA) and Kodac X‐ray films (abm, Cat no. B503).

### 2.14 Analysis of mRNA transcript levels

RNA was extracted from cells using Nucleospin RNA Plus columns (Macherey‐Nagel, Cat no. 740984) and cDNA was generated from 1 µg total RNA using the Transcriptor First Strand cDNA Synthesis Kit (Roche, Cat no. 04896866001). RT‐qPCR was performed using Brillant III SYBR Green Mastermix (Agilent, Cat no. 600883) on a CFX Connect (Biorad) and analyzed on CFX Maestro (Biorad). mRNA transcript levels were normalized to housekeeping *GUSB* mRNA expression. Primers used for qPCR reactions are on (Table S1) and annealing temperature was set at 55°C. Ct values remained below a threshold of 35 and the 2^−ΔΔCt^ method was utilized for RNA expression analysis.

### 2.15 Statistical analysis

All results are the average of three independent manipulations, performed in duplicate. Graphics and statistical analyses were produced and performed on GraphPad Prism 5. Statistical significance was calculated using student's two‐tailed unpaired t test analyses when applicable.

## 3 RESULTS

### 3.1 CRISPR‐Cas9 screening procedure

**FIGURE 1:**
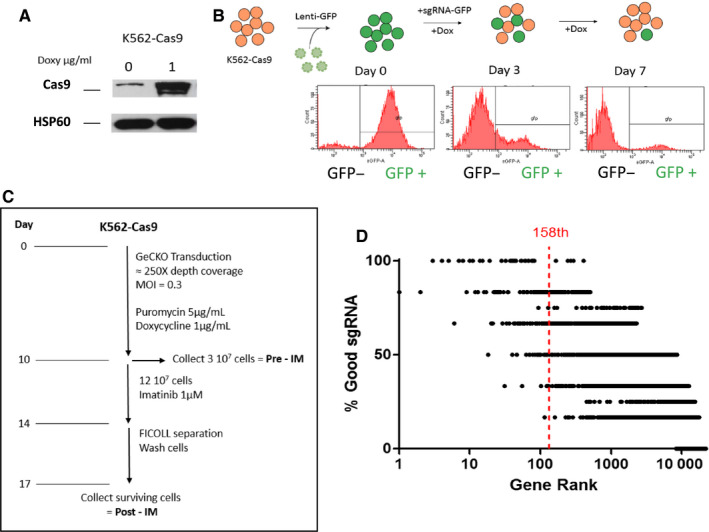
Generation of Cas9^+^ cell line and screening procedure. (A) K562 Cas9–positive cell lines express increased amounts of Cas9 endonuclease in the presence of 1 µg/mL doxycycline. (B) K562 Cas9^+^ cells were transduced with a GFP containing lentivirus. Cells were subsequently transduced with an sgRNA targeting the GFP gene. After 3 days of doxycycline induction, GFP levels were decreased in these cells, demonstrating efficient GFP knock‐out through CRISPR‐Cas9 activity. (C) K562 Cas9 cells were transduced with the GeCKO bank at a average coverage of approximately 250X and at an MOI = 0.3 to limit multiple sgRNA integrations in the same cell. sgRNA‐expressing cells were selected with puromycin and Cas9 expression was enabled with doxycycline. After 10 days, 3.10^7^ cells were collected as the control population, pre‐imatinib treatment. 12.10^7^ cells were subsequently treated to imatinib at 1 µmol/L for four days. Surviving cells were separated using FICOLL®, plated in fresh medium for 72 hours then collected as the imatinib treated population. (D) List of the percentage of good sgRNAs per gene in our generated rank. In the GeCKO library, protein targeting genes have 6 independent sgRNAs whereas miRNA have 4 independent sgRNAs

We performed a genome‐wide CRISPR‐Cas9 screen to select for genes involved in imatinib resistance in K562 cell lines. We created a clonal K562 cell line possessing a doxycycline‐inducible Cas9 (Figure 1A) and verified for Cas9 nuclease activity by performing a GFP cleavage assay (Figure 1B). GFP fluorescence was virtually extinguished after 7 days of Cas9 activity. We then transduced this K562 Cas9^+^ cell line with the GeCKO v2 library, as explained in materials and methods. Cells were then challenged to imatinib selection at 1 µmol/L for 4 days until ≈90% mortality was observed. This treatment protocol seems shorter and more drastic in comparison with other screening procedures that typically treat cells for a minimum of 2 weeks at lower drug concentrations.^3^ The goal was to prevent acquired CRISPR‐independent resistance to imatinib, as previously described,^22^ by treating the cells at a high imatinib concentration (1 µmol/L). DNA extraction of both pre‐ and postimatinib populations, sgRNA amplification by PCR, and next‐generation sequencing experiments were performed following the protocols in materials and methods (Figure 1C). We performed the screening process twice. After normalization, the coverage was similar in all samples (Figure S1). The enrichment of sgRNAs in both resistant populations compared to control populations was analyzed by MAGeCK (Table S2). Among all genes tested, 158 were enriched at an FDR (false discovery rate) <0.5 (or *P* value < .0036) and these top ranked genes had an enrichment of 73.7% ± 18.6% of the library's sgRNAs (four for miRNAs, or six for protein‐coding genes) (Figure 1D; Table S2).

### 3.2 Validation of the screening process through individual gene knock‐out in K562 cells

**FIGURE 2:**
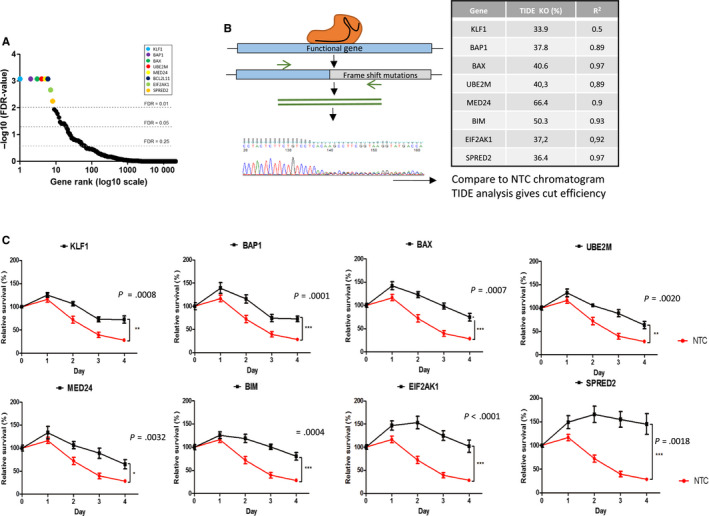
CRISPR screen validation. (A) Gene rank of all genes screened (20 914 different genes and miRNAs) according to their FDR‐value. Cut‐offs at FDR = 0.25, FDR 0.05 and FDR 0.01. (B) Individual gene knock‐out protocol: specific sgRNAs are cloned into lentiviral vectors and transduced into Cas9‐expressing cells. Targeted loci were subsequently Sanger sequenced. Indel efficiency is generated using TIDE analysis. (C) Proliferation of CRISPR modified cell lines and NTC cell line at 0.5 µmol/L during four days compared was analyzed by MTS and survival was calculated relative to day 0, n = 3. Student's two tailed unpaired *t*‐tests were performed on day 4 to calculate *P* values

In order to confirm the efficacy of the screening process, the top eight genes with an FDR <0.01 of the screen (*KLF1*, *BAP1*, *BAX*, *UBE2M*, *MED24*, *BIM*, *EIF2AK1,* and *SPRED2*) were targeted for individual CRISPR‐Cas9 gene knock‐out (Figure 2A). The most enriched sgRNA from the GeCKO library for each gene was individually cloned into lentiGuide‐Puro backbones. Ten days after transducing these vectors into K562 Cas9^+^ cells, the targeted loci were sequenced using Sanger sequencing and the CRISPR cut efficiencies were evaluated by TIDE analysis (Figure 2B). This software estimates the efficiency of CRISPR‐induced knock‐outs by determining the proportion of cells with frameshift mutations. All of the eight genes were knocked‐out at varying efficiencies (33.9%‐66.4%). These modified cell lines were then incubated in medium containing imatinib (0.5 µmol/L) and cell proliferation was tracked. All eight CRISPR modified cell lines showed decreased sensitivity to imatinib treatment over 4 days and had higher overall survival levels compared to the control cell line (transduced by a nontargeting control sgRNA [NTC]) (Figure 2C). This validates the screening process in vitro on K562 cells as individual gene knock‐out of the top hits does indeed affect sensitivity to imatinib.

### 3.3 Analysis of the function of enriched sgRNAs and gene sets in resistant cells

**FIGURE 3:**
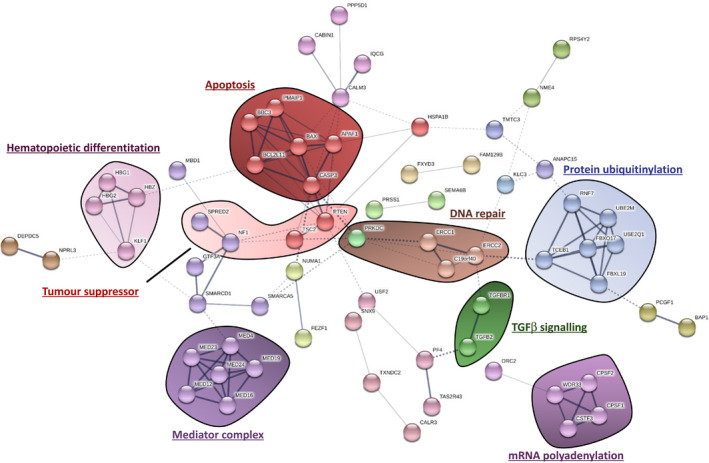
Functional protein association network analysis using STRING DB. The list of the top 158 genes identified through the CRISPR screen was inputted into STRING DB v10.5, unconnected nodes were excluded from the network. Multiple different cellular processes were identified using this approach

First, we focused our analysis on the top 158 hits (Table S3). STRING DB functional protein association network analysis of these top genes unveiled multiple enriched biological processes that seem important for imatinib‐induced cell death (Figure 3), such as proapoptotic signaling, hematopoietic differentiation, tumor suppressor signaling, DNA damage repair, mRNA polyadenylation, protein ubiquitinylation, TGFβ signaling, and the mediator complex.

Second, we performed a more global analysis. Gene set enrichment analysis was used to further determine which cellular processes or components seem important for imatinib‐induced cell death by inputting the complete ranked list of genes. Additionally, we analyzed the enrichment score (ES) of control sgRNAs (NTC) in the ranked list and discovered that NTCs had a ES^low^ = 2827, meaning that all genes ranked above 2827 in our screening process seem to be, to some degree, enriched (MAGeCK *P* value < .054) (Figure S2A). GSEA analysis also revealed similar enriched processes (Figure S2B‐H) such as the mediator complex, intrinsic apoptosis, mRNA polyadenylation, calcium‐activated channels, protein ubiquitinylation, cell growth, and G protein–coupled receptors.

### 3.4 The importance of intrinsic apoptosis in response to TKI treatment

Intrinsic apoptosis is a fine‐tuned mechanism that takes into account the balance of multiple different pro‐ and antiapoptotic proteins. Proapoptotic proteins are divided into three categories: activators, sensitizers, and effectors. Activators (BIM and BID) induce the dimerization of effectors (BAX and BAK) to form the MOMP (mitochondrial outer membrane permeabilization) and prime the mitochondria for cytochrome c release. This mechanism is also controlled positively by sensitizers (BAD, NOXA, PUMA), whose role is to target and inhibit antiapoptotic proteins such as BCL‐2 and MCL‐1 (Figure S3). These antiapoptotic proteins act by sequestering proapoptotic proteins by binding to their BH3 domain. GSEA and STRING DB analyses of the MAGeCK rank shows that the intrinsic apoptosis pathway is highly important for imatinib‐induced cell death in K562 cells (Figure S2C). In fact, the activator BIM (BCL2L11) (rank: 6th), the effector BAX (rank: 2nd), the sensitizers PUMA (rank: 71st) and NOXA (rank: 22nd), and other downstream proapoptotic proteins (APAF1 [rank: 13^th^] and CASP3 [rank: 47^th^]) are all highly ranked in the imatinib resistance screen, further demonstrating the crucial role of this cellular process with regard to response to treatment. BID, BAK, and BAD were not significantly enriched is the screening process (10 532nd, 1910th and 15 095th, respectively), perhaps suggesting a slightly lesser role of these genes with regard to imatinib resistance in CML.

### 3.5 The role of BIM and BAX with regard to BCL‐2 antagonism with BH3 mimetics

**FIGURE 4:**
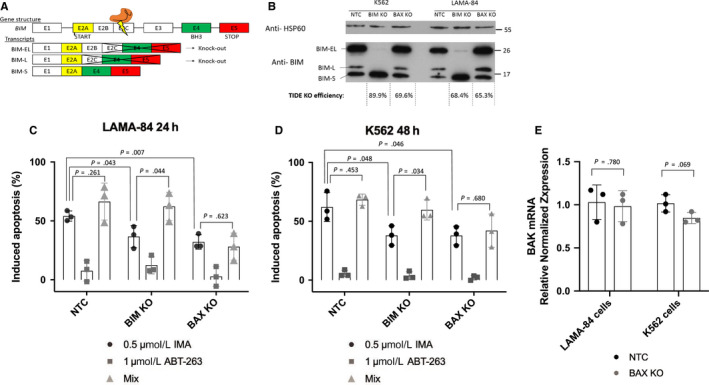
The importance of intrinsic apoptosis in response to TKI treatment. (A) Gene structure of *BIM*, CRISPR targeted exon 2C, knocking out BIM‐EL and BIM‐L isoforms. (B) Western blot analysis of HSP60 and BIM, knock‐out efficiency calculated with TIDE analysis software. (C, D) Induced apoptosis was calculated for all cell lines after either 24 hours (LAMA‐84) or 48 hours (K562) treatment with 0.5 µmol/L imatinib (IMA), 1 µmol/L navitoclax (ABT‐263) or both (Mix). (E) *BAK* transcript levels were determined by RT‐qPCR. Expression is normalized to housekeeping gene *GUSB*. Statistical analysis performed on GraphPad, student's two tailed unpaired *t*‐tests were performed, n = 3 biological replicates

Mutations and polymorphisms in proapoptotic genes *BIM* and *BAX* have been correlated to resistance to treatment in many cancer types, including CML.^23^, ^24^ To study these genes, we performed more efficient knock‐outs (68.4%, *r* ^2^ = 0.83 and 89.9%, *r* ^2^ = 0.95 for LAMA‐84 and K562 cells, respectively) of *BIM* with the previously utilized sgRNA for *BIM* KO (Figure 2B,C), which targets the exon 2C of the gene (Figure 4A). This targeted mutagenesis resulted in a depletion of the active isoforms, BIM‐EL and BIM‐L in the two cell lines, without affecting the less active BIM‐S^25^ (Figure 4B; Figure S5). We noticed that the BIM‐S isoform was significantly increased in cells lacking BIM‐EL and BIM‐L, suggesting a potential activation feedback loop between the isoforms. The levels of BIM isoforms are not significantly increased in *BAX* KO cells, demonstrating no compensatory role between the two apoptotic genes. Additionally, the residual levels of BIM‐EL that can be observed in LAMA‐84 *BIM* KO cells are due to the knock‐out efficiency being only at 68.4%.

To study *BAX* depletion, we equally performed more efficient knock‐outs (69.6%, *r* ^2^ = 0.96 and 65.3%, *r* ^2^ = 0.92 for K562 and LAMA‐84 cells, respectively) of *BAX* by utilizing the same sgRNA as the previous *BAX* KO analysis (Figure 2B,C), which targets the exon 3 of the gene, exon which contains the BH3 domain.

As expected, both *BIM* KO and *BAX* KO cell lines show increased resistance to imatinib treatment, observed by annexin V/propidium iodide labeling (Figure 4C). We then challenged the cells to BH3‐mimetic treatment (ABT‐263 or navitoclax) in an attempt to induce apoptosis and reverse resistance phenotypes. Briefly, ABT‐263 binds to two antiapoptotic proteins of the BCL‐2 family (both BCL‐2 and BCL‐x_L_), resulting in an efficient blockade of the inhibition of intrinsic apoptosis, thus releasing the proapoptotic BH3 domain containing proteins (such as BIM and BAX), and finally inducing apoptosis.^26^ Alone, ABT‐263 treatment shows low amounts of apoptotic induction (<20%), whatever the cell line. However, treating the cells with a combination of imatinib and ABT‐263 could only overcome the resistance phenotypes in the *BIM* KO cells, as previously observed,^27^ without affecting sensitivity to treatment in *BAX* KO cells (Figure 4C). These results were observed in two different cell lines, K562 and LAMA‐84. BAX expression seems essential for the activation of apoptosis by ABT‐263. This drug releases effectors BAX and BAK, which form mitochondrial outer membrane permeabilization (MOMP) channels and induce cytochrome c release and apoptosis. In our study, BAK alone is not sufficient for the induction of apoptosis in *BAX* KO cells. Additionally, no transcriptional compensation between these effectors was observed after RT‐qPCR analysis of *BAK* mRNA in the knock‐out cell lines (Figure 4D). We even observed a slight decrease of *BAK* transcription in K562 *BAX* KO cells.

A combination of imatinib and a BH3 mimetic may prove to be beneficial for resistant CML cells bearing mutations in *BIM*, but would seem ineffective on patients with lower levels of *BAX*, as BAK does not seem to compensate for the lack of BAX. Combining ABT‐263 to imatinib treatment would seem to have no effect for such resistance cells.

### 3.6 Targeting the MAPK pathway

Among the top hits in our CRISPR screen, SPRED2 (rank: 8th) stood out as a potential inhibitor of MAPK signaling.^28^ We equally sought to investigate the importance of the MAPK pathway with regard to imatinib resistance. Recently, it has been demonstrated that the inhibitory role of *SPRED2* on cell proliferation has been tightly correlated to the expression of the well‐known tumor suppressor *NF1*,^29^ equally highly ranked in the screen (rank: 12th). To further study the effects of MAPK activation and imatinib resistance, we generated K562 *SPRED2* KO and SPRED2 overexpression cell lines (*SPRED2* KO and SPRED2+, respectively) to investigate the potential therapeutic benefit of targeting MAPK signaling in CML *SPRED2* KO was obtained through dual sgRNA cleavage and efficient at 88.9%, whereas SPRED2 overexpression was obtained through lentiviral transduction.

**FIGURE 5:**
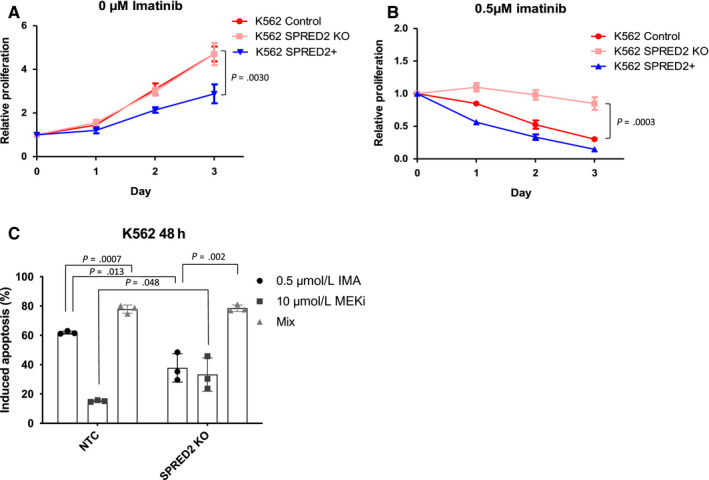
Targeting the MAPK pathway. Proliferation of K562 control, SPRED2 knock‐out (SPRED2 KO) and SPRED2 overexpressed (SPRED2+) was analyzed in medium with 0 µmol/L imatinib (A) or 0.5 µmol/L imatinib (B) over 3 days, n = 3. (C) Induced apoptosis was calculated for K562 NTC (control) and K562 SPRED2 KO cell lines avec 48 hours treatment with either 0.5 µmol/L imatinib (IMA), 10 µmol/L pimasertib (MEKi) or both (Mix). Statistical analysis performed on GraphPad, student's two tailed unpaired *t*‐tests were performed, n = 3 biological replicates

Firstly, SPRED2 overexpression has a negative effect on cell proliferation (Figure 5A) without the presence of imatinib and RT‐qPCR shows a decrease in MAPK signaling transcriptional targets^30^ (*ETV4*, *ETV5*, *PHDLA1*) in SPRED2‐overexpressing cells (Figure S6). *SPRED2* knock‐out does not affect cell proliferation in absence of imatinib treatment, even if MAPK signaling is increased in these cells (Figure 5A). As previously demonstrated, *SPRED2* KO cells show an increase in resistance to imatinib (0.5 µmol/L) which is observed by annexin V/propidium iodide labeling (Figure 5C) and cell proliferation (Figure 5B). Furthermore, combining imatinib treatment with MAPK inhibition, through MEK1/2 blockade with the antineoplastic compound pimasertib (MEKi),^31^ overcomes resistance phenotypes observed in *SPRED2* KO cells (Figure 5C; Figure S7). Interestingly, these *SPRED2* KO cells seem to be more sensitive to MAPK inhibition alone than control cells. This may be due to a form of dependence on MAPK signaling in these cells, and blocking this major pathway may be the cause of increased apoptosis.

## 4 DISCUSSION

Technological advances in the last decade, such as NGS and CRISPR‐Cas9 gene‐editing, have permitted laboratories around the world to decipher many novel physiological processes through efficient genome‐wide screening. The utilization of CRISPR‐Cas9 to perform genetic screens gives scientists more insight into physiological processes compared to previous shRNA screening techniques. Additionally, easy‐to‐use bioinformatic tools such as MAGeCK has democratized the analysis of complex NGS data, giving power to scientists with limited bioinformatic background. Furthermore, data resulting from CRISPR knock‐out screens differs from those provided by shRNA knock‐down screens,^32^ even though efficiencies between both technologies are similar.^33^

We utilized the GeCKO v2 library to screen for genes involved in imatinib resistance in CML. Our screening procedure was robust as a subset of the highly ranked genes (*BAX*, *BIM*, *BAP1,* and *SPRED2*) had previously been correlated to either CML progression, imatinib resistance, or resistance to alternative therapies in different cancer types.^24^, ^34^, ^35^, ^36^, ^37^ Gene set enrichment and STRING DB analyses unveiled cellular processes that seem crucial for optimal imatinib‐induced cell death such as genes involved in intrinsic apoptosis.

Targeting this form of programmed cell death has proved to be a powerful novel form of therapy for cancer.^38^ Clinical trials that utilize BCL‐2 inhibitors are becoming more and more prevalent and could potentially show exciting results as an efficient treatment for many different forms of blood cancer, such as chronic lymphocytic leukemia, acute myeloid leukemia and Non‐Hodgkin's lymphoma.^38^

We showed that BH3 mimetics could potentially be utilized to treat certain forms of primary resistance to imatinib in CML, as cotreating *BIM* KO cells lines with imatinib and ABT‐263 (navitoclax) overcame resistance phenotypes. An ongoing clinical trial (ClinicalTrials.gov #NCT02689440) is currently assessing the efficacy of dasatinib combined with another BH3 mimetic, venetoclax, as first line therapy for early phase BCR‐ABL1 positive chronic myeloid leukemia.

Additionally, this treatment could show increased effectiveness for patients bearing *BIM* polymorphisms. The most common polymorphism in *BIM* is present in ≈21% of the East Asian population and results in an alternatively spliced mRNA, lacking the BH3 proapoptotic domain.^24^, ^39^ This mutation seems to cause resistance to treatment in different types of lung cancer,^39^ but its effect of imatinib resistance in the clinic is still questioned.^24^ For such resistant patients, navitoclax and imatinib combined could have more beneficial effects. A certain polymorphism in the promoter of the *BAX* gene G(‐248)A, has equally been correlated to increased resistance to treatment.^23^ However, our work suggests that BAX, a crucial protein involved in MOMP formation, is essential for BH3‐mimetic function and such therapies could possibly turn out to be ineffective for patients with decreased BAX function.

Along with targeting intrinsic apoptosis, we equally investigated the role of MAPK activation with regard to imatinib resistance. We decided to study the SPRED2 tumor suppressor to discover its role with regard to MAPK inhibition. SPRED2, a member of the Sprouty‐related family, had previously been associated as a potential negative prognostic marker in multiple malignancies such as CML,^34^ prostate cancer,^40^ and hepatocellular carcinoma.^41^ Its role as an inhibitor of MAPK signaling was further demonstrated in our work and specifically inhibiting this pathway with MEK inhibitor pimasertib overcame imatinib resistance phenotypes. Targeting this pathway may be beneficial as the MAPK pathway is a major survival and proliferation activator. We have already reported a synergistic inhibitory effect between navitoclax and pimasertib in acute myeloid leukemia.^42^

The regulation of additional BCR‐ABL1 related pathways such as PI3K/AKT/mTOR or Jak/STAT5 signaling may also play an important role in imatinib resistance. Among the top ranked genes, tumor suppressor *PTEN* knock‐out was highly associated to imatinib resistance (rank: 40th). Also, mTOR negative regulators *TSC1* and *TSC2* were equally highly ranked (237th and 26th, respectively) in the screening process. Targeting the PI3K signaling pathway with FDA‐approved compounds such as idelalisib (PI3K inhibitor) or everolimus (mTOR inhibitor) may also seem beneficial for imatinib‐resistant patients that show overactivation of the PI3K signaling pathway.^43^, ^44^

As previously indicated, a subset of the top‐ranking genes had already been correlated to imatinib resistance and CML progression. However, additional genes or cellular processes caught our interest. Another intriguing result that came from the CRISPR screening procedure was the enrichment of many Mediator complex subunits. The Mediator is a highly conserved eukaryotic multiprotein transcriptional coactivator complex.^45^ It is composed of around 26 different subunits, of which 10 (Figure S4) with *P* values < .05, are in the top 852 genes of our ranking (852nd ranked gene (*MED18*) has an enrichment *P* value = .035). Half of these enriched MED subunits are located in the tail of the Mediator complex, the regulatory subunit of the complex. Interestingly, MED16, MED23, and MED24 form a tight‐knit submodule in the tail of the mediator complex and becomes unstable and subject to degradation if one of the three subunits is deficient.^45^, ^46^ All three genes are highly ranked in our screen, meaning that this submodule could play an important role in imatinib‐induced cell death. Interestingly, four (MED12, MED16, MED19, and MED23) out of six of the mediator complex subunits found to be significantly enriched in our study were also enriched in the GeCKO screen performed by Shalem et al, who screened for verumafenib resistance (BRAF V600E inhibitor) in melanoma.^3^ This complex transcriptional regulator may be important for the induction of cell death after cancer cell inhibition through targeted therapy. Moreover, the MED16/MED23/MED24 submodule may potentially have a role in the transcriptional activation of apoptotic genes in response to cellular stress.^47^ It has been shown that MED23 knock‐down promotes tumorigenicity and inhibits apoptosis through a decrease of BAX expression, yet conversely, MED24 knock‐down in breast carcinoma cells lead to diminished cell proliferation and DNA synthesis.^46^ These contradictory results represent the complexity and diverse functionalities of the dynamic multiprotein Mediator complex.

Other cellular processes seem important for imatinib‐induced cell death in our model. However, their potential roles concerning resistance to treatment are currently not understood. Genes involved in protein ubiquitinylation and mRNA polyadenylation, for example, are significantly knocked‐out in imatinib resisting cells. Protein turnover through the regulation of gene transcription and protein degradation may be an important process for imatinib‐induced cell death.^48^, ^49^ In particular, *UBE2M*, was highly ranked in both imatinib resistance (rank: 8th) and verumafenib resistance screens (rank: 45th and 95th). This E3‐ubiquitinylating protein may have an important role with regard to protein turnover and drug resistance.

Understanding the genetics behind certain neoplasms or resistance phenotypes brings us one step closer to truly personalized medicine. Combining multiple FDA‐approved compounds for optimal response to treatment is becoming more and more favored in oncology. Patient's mutational burden and genotypes can vary drastically for all cancer types which may decrease prognostic values and increase the probability of drug resistance. Personalizing treatment according to patient's mutations may resolve issues regarding cancer persistence and resistance to treatment. In this study, we show that two possible combinations, imatinib and apoptotic inhibitor navitoclax or imatinib and MAPK inhibitor pimasertib, can overcome resistance to imatinib in specific cases in vitro. We also discussed many additional possibilities for targeting specific cancer pathways or drivers. Additional research needs to be performed to fully understand oncogenic transformation. However, advances in tumor sequencing, big data analysis, and machine learning will give future oncologists the best tools to find the most effective treatment combinations according to their patient's mutations and help advance the domain of precision oncology.

## CONFLICT OF INTEREST

The authors declare no potential conflicts of interest.

## AUTHOR CONTRIBUTION

ML, VPM, FL, RI, BT, and FXM conceived and planned the experiments. ML, VPM, and ER carried out the experiments. ML performed data analysis and ML, VPM, FL, RI, BT, and FXM contributed to the interpretation of results. ML took the lead in writing the manuscript and all authors provided critical feedback and helped shape the research, analysis, and manuscript.

## Supporting information

Supplementary Material

## Data Availability

Data available on request from the authors.
