# Supplementary material for: A genome‐scale CRISPR knock‐out screen in chronic myeloid leukemia identifies novel drug resistance mechanisms along with intrinsic apoptosis and MAPK signaling
Source: Cancer Med. 2020 Jul 28;9(18):6739–51. doi: 10.1002/cam4.3231 (PMC7520295; doi:10.1002/cam4.3231)
Supplement: Supplementary file 1 — Supplementary Material [file CAM4-9-6739-s001.pdf]

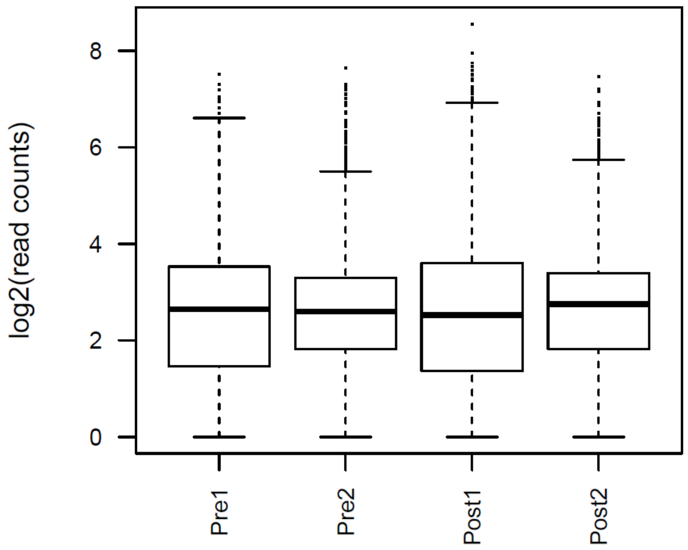

Supplementary Figure S1: Normalized read counts.
Log2 of normalized read counts after next generation sequencing of both pre-imatinib (Pre1, Pre2) and post-imatinib (Post1, Post2) populations.

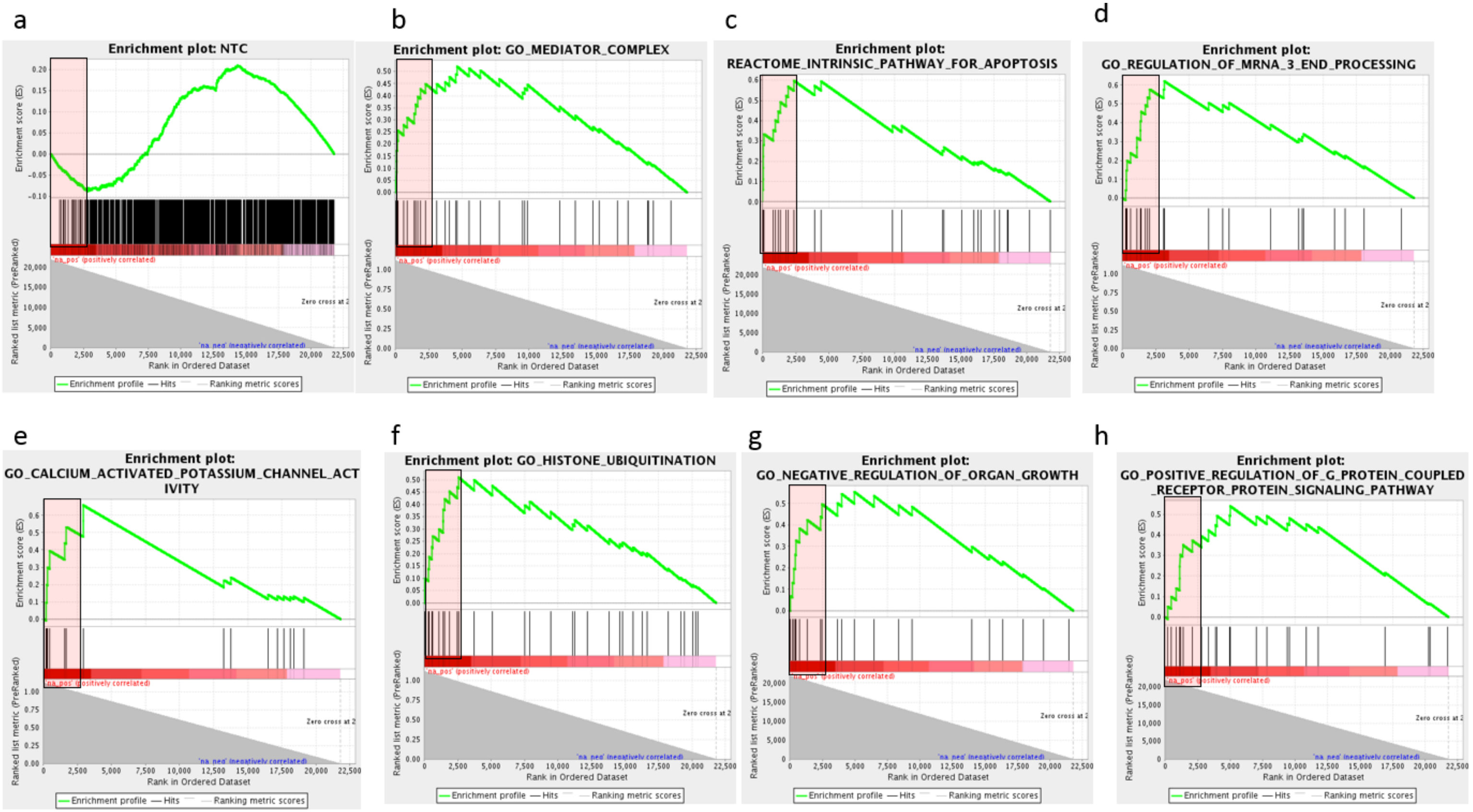

Supplementary Figure S2: Gene set enrichment analysis of entire gene rank.

GSEA was performed using the GSEA Preranked module as described in materials and methods.

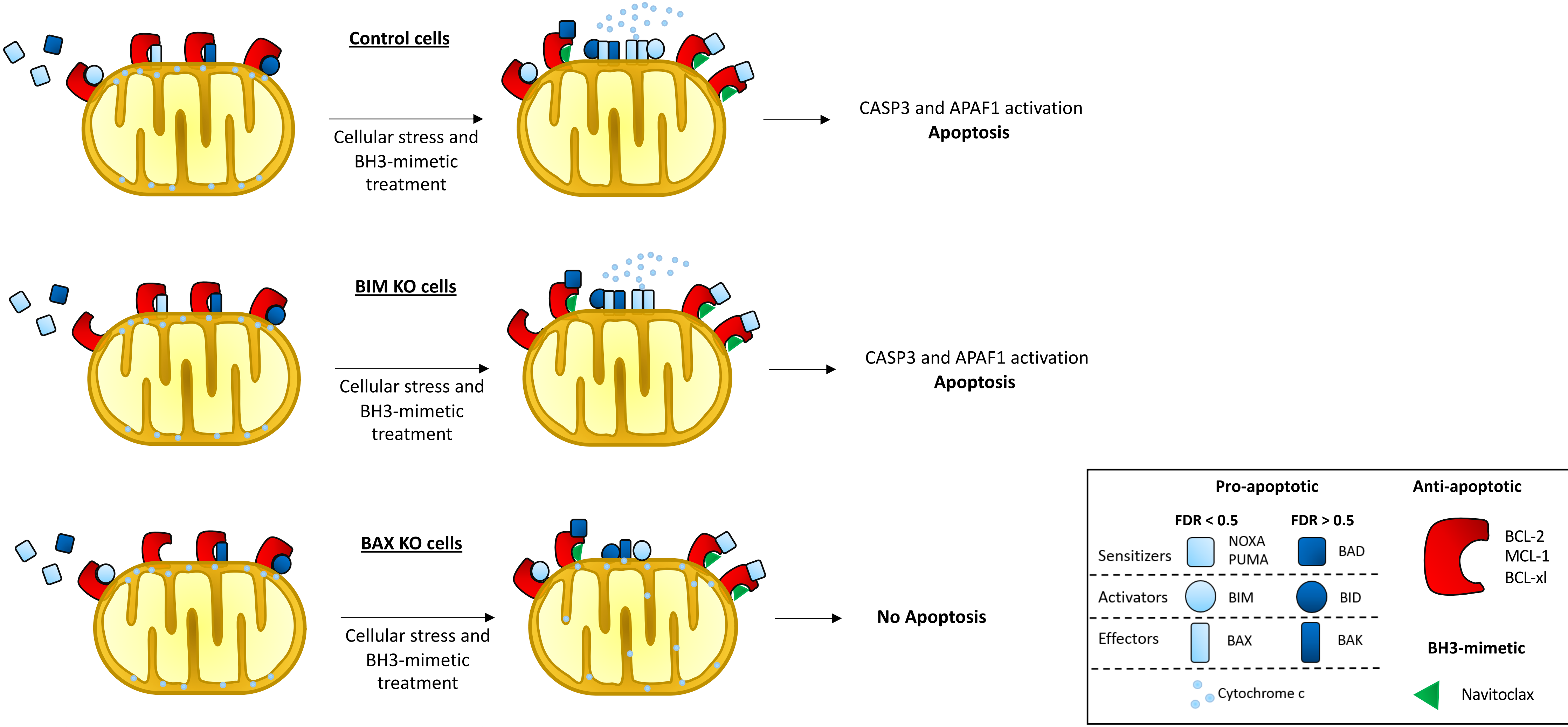

Supplementary Figure S3: Intrinsic apoptosis and genes enriched in CRISPR screen.
Proapoptotic proteins are classified into two subclasses, whose that are enriched in the imatinib resistance screen (light blue, FDR < 0.5) and those that are not (dark blue, FDR > 0.5). In control cells, cellular stress or BH3-mimetic treatment dislodges anti-apoptotic proteins from proapoptotic proteins, allowing cytochrome release and apoptosis. Similar phenotypes are observed in BIM KO cells. However, less apoptosis is observed in response to cellular stress or BH3-mimetic treatment in BAX KO cells. BAX seems essential for MOMP formation and cytochrome c release.

Supplementary Figure S4: The Mediator complex

Blue subunits are part of the tail, red subunits are part of the middle, green subunits are part of the head and yellow subunits are part of the CDK8 module. Subunits in the darker shade of the color are enriched in the imatinib resistance screen. The CRISPR screen rank beside the subunit. MED24, MED16 and MED23 compose a tight-knit submodule in the tail of the mediator complex.

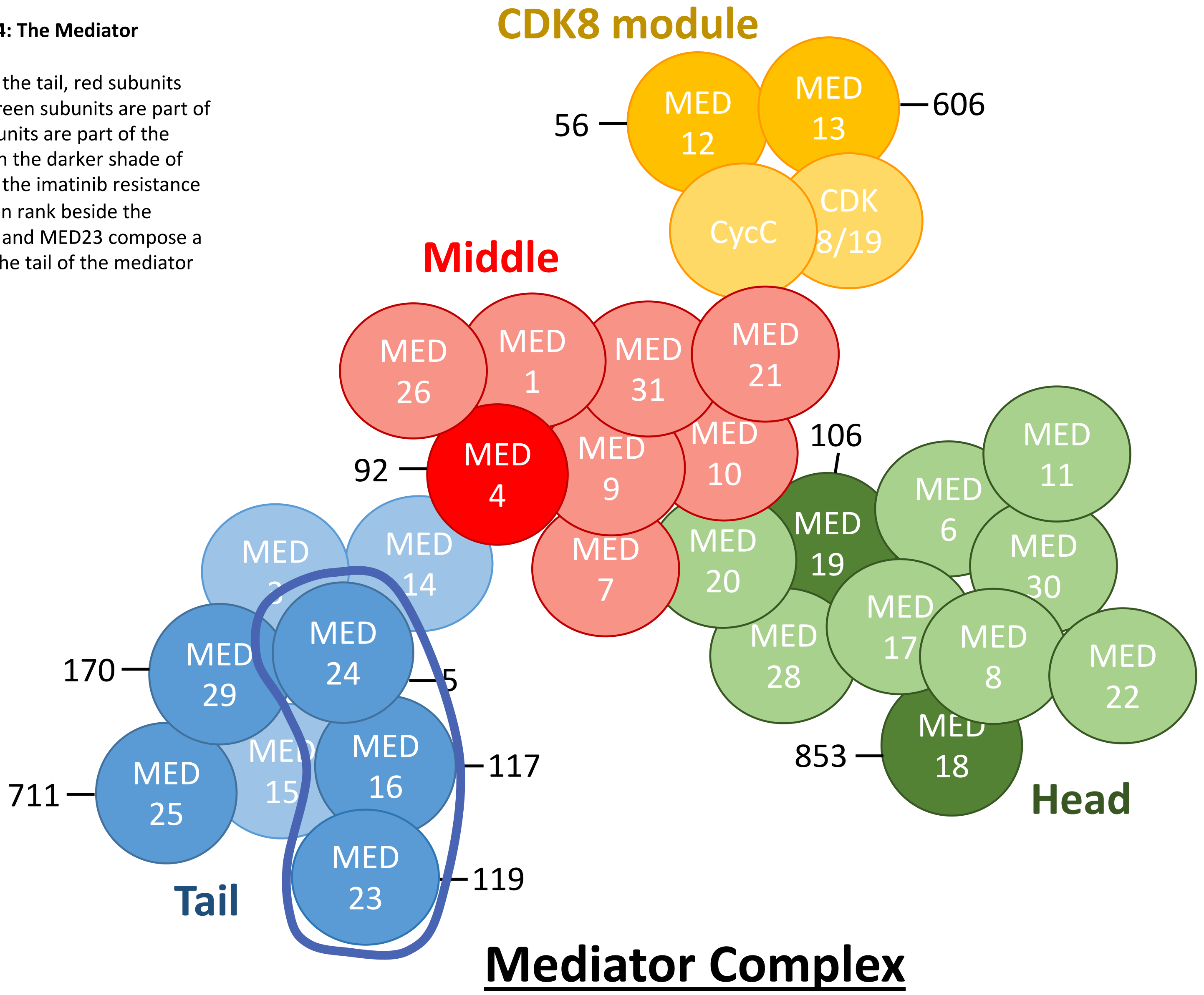

Supplementary Figure S5: Uncropped western blot analysis.
Top uncropped image was used for figure 4b to show actin staining (dotted red line). The bottom uncropped image was used for figure 4b to show BIM(EL, L and S) staining. Both images originate from same gel.

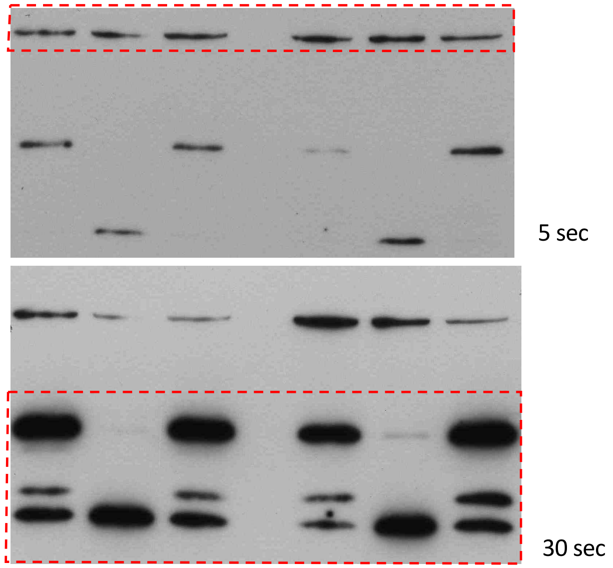

Supplementary Figure S6: Ras activation via RT-qPCR

Ras activation in SPRED2 KO cells was confirmed by RT-qPCR analysis. Statistical analysis performed on GraphPad, Student's two tailed unpaired t-tests were performed, n = 3 biological replicates

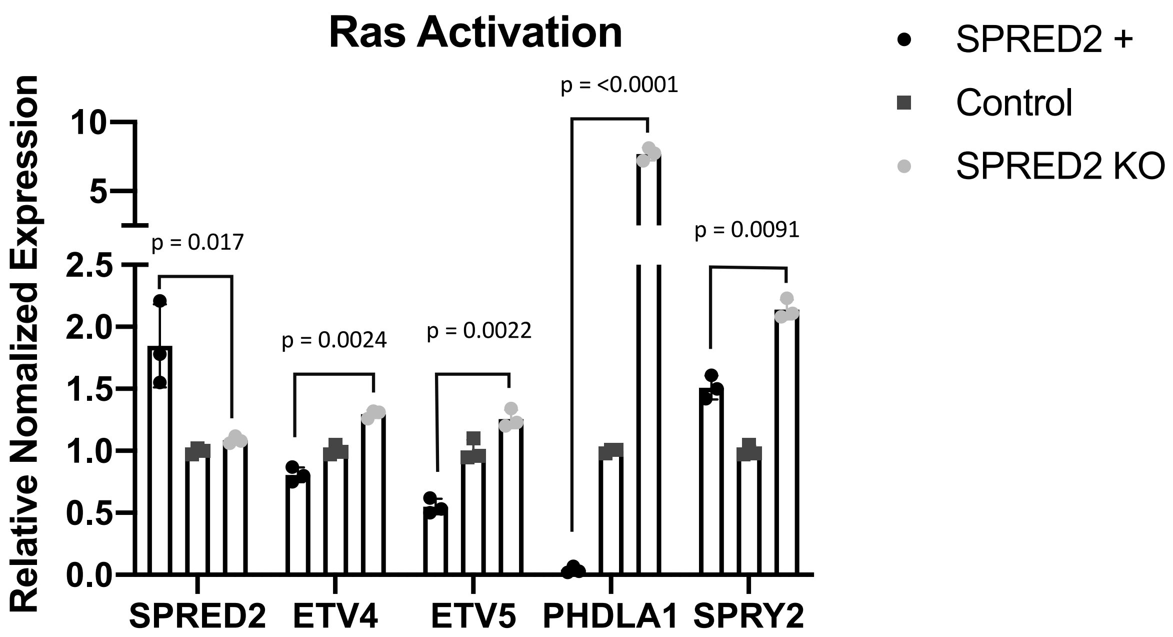

Supplementary Figure S7: FACS representative plots

Plots and gating strategy is shown. The % of DAPI+ cells was added to the % of Annexin V+ cells to calculate the overall % apoptosis. P1 (total population), DAPI (Dapi stained cells) and Annexin V pos (annexin V positive cells).

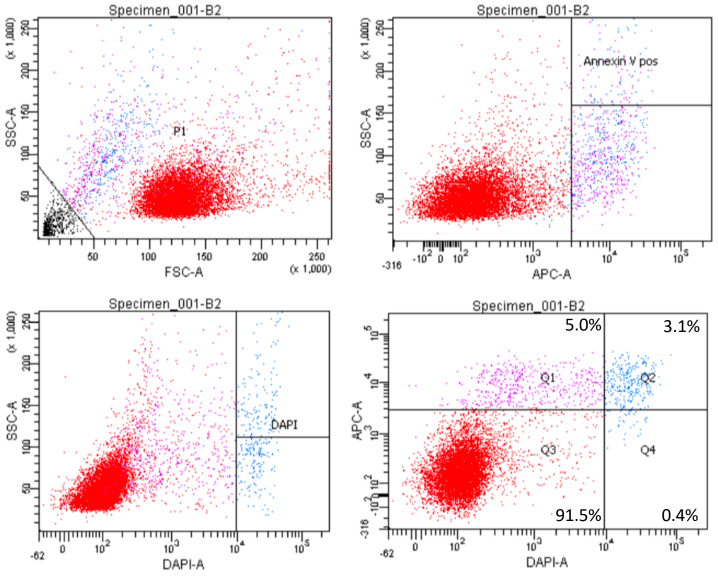

Untreated sample

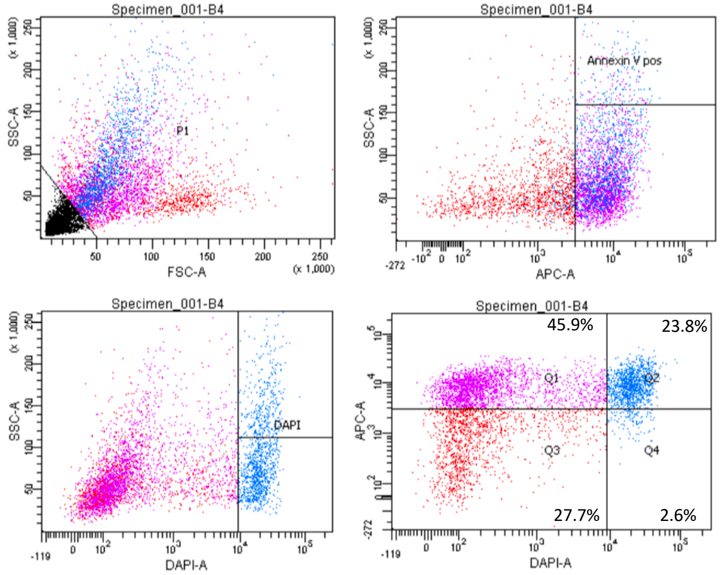

Treated sample

Supplementary table 1: Primers used for sgRNA cloning, PCR, Sanger and qPCR reactions

		Gene target	Primer ID	sgRNA cloning primers<br>Sequence 5' - 3'	CRISPR-targeted loci PCR primers and SANGER seq primers<br>PCR cut check F	PCR cut check R
		KLF1	sgRNA F KLF1<br>sfRNA R KLF1	caccgTCAGAGCGCGAAAAAGCACG<br>aaacCGTGCTTTTTCGCGCTCTGAc	TCAGTACCAAGGGCACTTCC	GAGTCCAGGAGAGGGTCCAT
	**BAX study**	BAX	sgRNA F BAX<br>sfRNA R BAX	caccgCCATTCGCCCTGCTCGATCC<br>aaacGGATCGAGCAGGGCGAATGGc	TCAGGGGTGAGTTTGAGGTC	GCTGGACTTCTGGGTCCTC
		BAP1	sgRNA F BAP1<br>sfRNA R BAP1	caccgCCAAGGTAGAGACCTTTCGC<br>aaacGCGAAAGGTCTCTACCTTGGc	CATCACCCCCTCCTCTTGT	CCTGTCTTCCTCCATTTCCA
		UBE2M	sgRNA F UBE2M<br>sfRNA R UBE2M	caccgTCACCAAGAAGAGATACTGC<br>aaacGCAGTATCTCTTCTTGGTGAc	CAGGCACCTACCCATCTCTC	TCCTTGTTCAGTGGGTCCTC
**Top 8 Check**	**BIM study**	BIM	sgRNA F BIM<br>sfRNA R BIM	caccgCAACCACTATCTCAGTGCAA<br>aaacTTGCACTGAGATAGTGGTTGc	CAACACAAACCCCAAGTCCT	CACATCCTCCATTCCCATCT
		MED24	sgRNA F MED24<br>sfRNA R MED24	caccgATACCTTACTGATGGCTGTG<br>aaacCACAGCCATCAGTAAGGTATc	TCCTTGCTTGGGTTGAAGAG	GCAATGCTTATGGGGAGAAA
		EIF2AK1	sgRNA F EIF2AK1<br>sfRNA R EIF2AK1	caccgAAACCCACTTCGTTCAAGAC<br>aaacGTCTTGAACGAAGTGGGTTTc	TTGCAAACCAACTCTTGCTG	ACCCGGCTAATTTCCATATTTT
		SPRED2	sgRNA F SPRED2<br>sfRNA R SPRED2	caccgCTGATGCCCGAGCCTTTGAC<br>aaacGTCAAAGGCTCGGGCATCAGc	GCCTGTGTGTTTCCATCCTT	TACTGACCTGGTCCCAAACC
		NTC	sgRNA F NTC<br>sfRNA R NTC	caccgATATTTCGGCAGTTGCAGCA<br>aaacTGCTGCAACTGCCGAAATATc		

	Gene target	Primer ID	Sequence 5' - 3'	PCR cut check F	PCR cut check R
**SPRED2 study**	SPRED2 A	sgRNA F SPRED2 A<br>sfRNA R SPRED2 A	caccgATGACCAGAGATGACTCCAG<br>aaacCTGGAGTCATCTCTGGTCATc	TGGTCATCTTGCATTTTGAAAC	CACGAGTTGTATGTCTGCCATT
	SPRED2 B	sgRNA F SPRED2 B<br>sfRNA R SPRED2 B	caccgGCCGCTTCGTCCATTGCCTT<br>aaacAAGGCAATGGACGAAGCGGCc		

U6 promoter	U6 F	CAAGGCTGTTAGAGAGATAATTGGA

	Gene target	Primer ID	Sequence 5' - 3'
**qPCR**	GUSB	GUSB F<br>GUSB R	GTCTGCGGCATTTTGTCGG<br>CACACGATGGCATAGGAATGG
	SPRED2	SPRED2 F<br>SPRED2 R	CTCATCCATGGTGAACGACAGAA<br>TGTCAAAGGCTCGGGCATC
	ETV4	ETV4 F<br>ETV4 R	CAGTGCCTTTACTCCAGTGCC<br>CTCAGGAAATTCCGTTGCTCT
	ETV5	ETV5 F<br>ETV5 R	CAGTCAACTTCAAGAGGCTTGG<br>TGCTCATGGCTACAAGACGAC
	PHDLA1	PHDLA1 F<br>PHDLA1 R	GAAGATGGCCCATTCAAAAGCG<br>GAGGAGGCTAACACGCAGG
	SPRY2	SPRY2 F<br>SPRY2 R	CCTACTGTCGTCCCAAGACCT<br>GGGGCTCGTGCAGAAGAAT
	BAK	BAK F<br>BAK R	ATGGTCACCTTACCTCTGCAA<br>GAGTCATAGCGTCGGTTGATG

Supplementary Table 2: Ranked list of genes enriched in imatinib resistant cells

gene id	number of sgF	adj-RRA score	p-value	FDR	rank	#goodsgrna
KLF1	6	3.6479e-09	2.2693e-07	0.000825	1	5
BAX	6	7.4752e-09	2.2693e-07	0.000825	2	5
BAP1	6	1.4198e-08	2.2693e-07	0.000825	3	6
EIF2AK1	6	4.2378e-08	2.2693e-07	0.000825	4	6
MED24	6	5.8985e-08	2.2693e-07	0.000825	5	6
BCL2L11	6	7.4622e-08	2.2693e-07	0.000825	6	4
SPRED2	6	1.2229e-07	6.8079e-07	0.002122	7	6
UBE2M	6	3.1071e-07	2.0424e-06	0.005569	8	6
SMARCA5	6	5.9396e-07	4.7655e-06	0.011551	9	5
DMRTC2	6	8.9804e-07	6.1271e-06	0.013366	10	6
TGFBR1	6	1.4203e-06	8.3964e-06	0.016652	11	6
NF1	6	2.467e-06	1.2481e-05	0.02269	12	5
APAF1	6	3.8832e-06	2.0197e-05	0.033892	13	5
SLC30A1	6	5.1204e-06	2.6551e-05	0.03873	14	6
hsa-mir-142	4	5.3955e-06	2.7912e-05	0.03873	15	4
TMEM163	6	5.7022e-06	2.9274e-05	0.03873	16	5
FBXO17	6	5.9941e-06	3.0182e-05	0.03873	17	5
TARBP2	6	7.823e-06	3.699e-05	0.044829	18	3
BLVRB	6	8.7407e-06	4.2436e-05	0.048723	19	6
WDR87	6	1.0856e-05	5.2875e-05	0.057673	20	4
SNX9	6	1.2247e-05	6.1498e-05	0.063885	21	4
PMAIP1	6	1.6644e-05	8.1014e-05	0.080333	22	5
KPTN	6	1.8857e-05	9.5084e-05	0.090185	23	5
RREB1	6	1.9982e-05	9.9623e-05	0.090553	24	5
HBZ	6	2.4157e-05	0.00012368	0.107921	25	5
TSC2	6	2.5113e-05	0.00012912	0.10834	26	5
NPRL3	6	3.4526e-05	0.00017406	0.136053	27	5
DEPDC5	6	3.5515e-05	0.0001795	0.136053	28	6
SMARCD1	6	3.5915e-05	0.00018086	0.136053	29	4
ORC2	6	3.8076e-05	0.00019221	0.139769	30	5
ZNF341	6	4.4615e-05	0.00022489	0.153611	31	2
CXCL17	6	4.562e-05	0.0002317	0.153611	32	6
PCGF1	6	4.7078e-05	0.00023805	0.153611	33	4
RALGAPA2	6	4.7768e-05	0.00023941	0.153611	34	5
ERCC2	6	5.0253e-05	0.00025121	0.156577	35	5
YIF1B	6	5.4125e-05	0.00027027	0.163497	36	4
WDR33	6	5.728e-05	0.00028298	0.163497	37	6
USP22	6	5.7603e-05	0.0002848	0.163497	38	6
hsa-mir-6888	4	6.0495e-05	0.00029978	0.163608	39	4
PTEN	6	6.1477e-05	0.00030204	0.163608	40	3
TXNDC2	6	6.2336e-05	0.00030749	0.163608	41	6
PPP5D1	6	6.8162e-05	0.00033518	0.171656	42	4
RGS11	6	6.8793e-05	0.00033835	0.171656	43	6
MDN1	6	7.1363e-05	0.00034879	0.17293	44	4
CPSF1	6	7.9961e-05	0.00039645	0.19024	45	6
TAS2R43	6	8.3356e-05	0.00041006	0.19024	46	3
CASP3	6	8.513e-05	0.00042096	0.19024	47	4
PLGLB2	4	8.5804e-05	0.0004255	0.19024	48	4
FEZF1	6	8.6149e-05	0.00042731	0.19024	49	4

SSX7	6	9.2412e-05	0.00046226	0.201683	50	4
UBE2Q1	6	0.00010331	0.000514	0.21986	51	5
RPS4Y2	6	0.00011411	0.000573	0.239212	52	4
LRRCC1	6	0.00011564	0.00058117	0.239212	53	6
ERCC1	6	0.00011759	0.00059342	0.239732	54	6
LRRK1	6	0.00012556	0.00063291	0.251035	55	2
MED12	6	0.00012864	0.00064925	0.251536	56	4
CDCA7	6	0.00013098	0.00065787	0.251536	57	5
CCZ1B	4	0.00013342	0.00066877	0.251536	58	4
PSG5	6	0.00013931	0.00068964	0.254992	59	5
ESM1	6	0.00014286	0.00070825	0.256208	60	4
IRX4	6	0.00014445	0.00071642	0.256208	61	6
USF2	6	0.00014847	0.0007314	0.257346	62	6
PRKDC	6	0.0001524	0.00075273	0.260647	63	3
GSTCD	6	0.00016001	0.00078586	0.267868	64	5
TCEB1	6	0.00017319	0.00085666	0.283213	65	4
ZNF235	6	0.0001752	0.00086756	0.283213	66	5
ZNF382	6	0.00017563	0.00086983	0.283213	67	6
CYP2A13	6	0.0001821	0.00090568	0.29055	68	6
FAM159A	6	0.00019115	0.00095107	0.300689	69	3
DPY19L3	6	0.00020839	0.0010405	0.324257	70	4
BBC3	6	0.00021521	0.0010763	0.330707	71	3
RBM44	6	0.00023046	0.0011508	0.333115	72	4
NME4	6	0.00023304	0.001163	0.333115	73	5
GAREM	6	0.00023425	0.0011689	0.333115	74	5
TACO1	6	0.00023765	0.0011857	0.333115	75	6
LIPG	6	0.00023876	0.0011907	0.333115	76	6
C11orf35	6	0.00023998	0.0011966	0.333115	77	4
CC2D1A	6	0.00024408	0.0012148	0.333115	78	3
C1QTNF3	6	0.00025252	0.0012547	0.333115	79	5
PF4	5	0.00025433	0.001262	0.333115	80	5
KCTD5	6	0.00025459	0.0012633	0.333115	81	6
HSPA1B	6	0.00025512	0.0012665	0.333115	82	6
RBAK-LOC3894	3	0.00025531	0.0012674	0.333115	83	3
FSTL3	6	0.00027069	0.0013282	0.342167	84	5
FXYD3	6	0.0002724	0.0013332	0.342167	85	4
MSMO1	6	0.00028519	0.0013868	0.346874	86	4
SMCHD1	6	0.00028662	0.0013922	0.346874	87	5
ZNF155	6	0.00029838	0.0014385	0.346874	88	4
CYP27A1	6	0.00030226	0.0014544	0.346874	89	3
PRSS1	6	0.00031246	0.0014989	0.346874	90	4
MED4	6	0.00031578	0.0015102	0.346874	91	3
LZTR1	6	0.00031624	0.0015125	0.346874	92	5
hsa-mir-6088	4	0.00032105	0.0015311	0.346874	93	3
SAMD4B	6	0.00032494	0.0015474	0.346874	94	5
ADIPOR2	6	0.00032642	0.001552	0.346874	95	3
KLC3	6	0.0003299	0.0015629	0.346874	96	4
TMEM67	6	0.0003317	0.0015729	0.346874	97	3
RUNX1T1	6	0.0003317	0.0015729	0.346874	98	5
ZNF585A	6	0.00033913	0.0016001	0.346874	99	5
MAOA	6	0.00034281	0.0016146	0.346874	100	3
DYSF	6	0.00034367	0.0016178	0.346874	101	2

HKR1	6	0.00034486	0.0016219	0.346874	102	5
DOK4	6	0.00036062	0.001699	0.359848	103	5
CNIH4	6	0.00037911	0.0017766	0.36518	104	4
MED19	6	0.00037973	0.0017789	0.36518	105	4
NEK9	6	0.00038209	0.0017884	0.36518	106	5
ADAM30	6	0.00038249	0.0017912	0.36518	107	5
TGFB2	6	0.00039157	0.0018248	0.368583	108	4
HBG1	5	0.00040053	0.0018579	0.371832	109	4
MBD1	6	0.00041675	0.0019223	0.373526	110	5
FAM129B	6	0.00041861	0.001931	0.373526	111	5
FAM217B	6	0.00041982	0.0019382	0.373526	112	3
DHX29	6	0.00042389	0.0019577	0.373526	113	4
FOXR1	6	0.00042674	0.0019686	0.373526	114	5
VPS13C	6	0.00042684	0.0019691	0.373526	115	1
HBG2	3	0.00043211	0.0019922	0.374659	116	3
MED16	6	0.00044372	0.0020308	0.378649	117	4
CPSF2	6	0.00045835	0.0020912	0.385681	118	5
MED23	6	0.00046159	0.0021039	0.385681	119	4
MLF2	6	0.00047705	0.0021633	0.39068	120	3
FBXL19	6	0.00047793	0.002167	0.39068	121	4
UNC119B	6	0.00049206	0.002226	0.398028	122	2
AIDA	6	0.0005016	0.0022595	0.400749	123	5
VPS13D	6	0.00052725	0.0023571	0.411394	124	2
NUMA1	6	0.00053103	0.0023739	0.411394	125	3
DHRS9	6	0.00053287	0.0023835	0.411394	126	4
SEMA6B	6	0.00053645	0.0023998	0.411394	127	5
CABIN1	6	0.00054026	0.0024139	0.411394	128	4
hsa-mir-4742	4	0.00056282	0.0025119	0.422259	129	3
CLEC2D	6	0.0005707	0.0025468	0.422259	130	2
CSTF3	6	0.0005738	0.0025582	0.422259	131	4
ARHGAP35	6	0.00057746	0.0025723	0.422259	132	3
ABHD17B	6	0.00058218	0.0025895	0.422259	133	3
SORCS2	6	0.0005885	0.0026122	0.422259	134	3
TTC9B	6	0.00058854	0.0026131	0.422259	135	5
CRYBB3	6	0.00059495	0.0026417	0.423741	136	5
RCE1	6	0.00060062	0.0026667	0.424622	137	5
CD2	6	0.00063356	0.0028037	0.443213	138	4
C19orf40	6	0.00064964	0.0028659	0.449783	139	5
ZC3HC1	6	0.00066433	0.0029213	0.450669	140	5
RNF7	6	0.00066551	0.0029254	0.450669	141	4
GTF3A	6	0.00066816	0.0029335	0.450669	142	3
MFSD9	6	0.00067785	0.0029717	0.453334	143	3
ZNF99	6	0.00070094	0.0030661	0.464487	144	5
BIN2	6	0.00070828	0.0030924	0.465244	145	3
CMTM8	6	0.00073646	0.003204	0.473221	146	2
CALR3	6	0.0007414	0.0032199	0.473221	147	5
TMTC3	6	0.00074328	0.0032285	0.473221	148	4
IQCG	6	0.00074472	0.0032322	0.473221	149	4
hsa-mir-4799	4	0.00078664	0.0033783	0.486223	150	2
GABPB2	6	0.00078824	0.0033838	0.486223	151	4
LPPR1	6	0.0007893	0.0033878	0.486223	152	5
CALM3	6	0.00081456	0.0034809	0.49386	153	5

CILP	6	0.00081646	0.0034863	0.49386	154	5
ANAPC15	6	0.00082844	0.003539	0.497838	155	4
IAH1	6	0.0008335	0.0035621	0.497838	156	4
hsa-mir-548f-3	3	0.0008431	0.0036057	0.497838	157	3
SHPK	6	0.00084321	0.0036057	0.497838	158	4
FZD6	6	0.00087863	0.0037546	0.513424	159	1
TAF13	6	0.00088281	0.0037709	0.513424	160	3
SLC45A2	6	0.0008909	0.0038	0.513424	161	5
ZNF599	6	0.00089702	0.0038231	0.513424	162	2
ESRP2	6	0.00090045	0.0038363	0.513424	163	4
LYPD4	6	0.00090862	0.0038626	0.513795	164	4
PRIMA1	6	0.00092881	0.0039461	0.516885	165	2
hsa-mir-5100	4	0.00093428	0.003967	0.516885	166	4
SDR9C7	6	0.00093545	0.0039729	0.516885	167	2
JMJD1C	6	0.00093739	0.0039806	0.516885	168	5
MED29	6	0.00097116	0.0041036	0.52383	169	5
LGALS9	6	0.000979	0.0041317	0.52383	170	2
hsa-mir-346	4	0.00098215	0.0041394	0.52383	171	3
STAG2	6	0.00098908	0.0041621	0.52383	172	3
HBE1	6	0.00099709	0.0041912	0.52383	173	4
SBSN	6	0.00099998	0.0042034	0.52383	174	3
SLC27A3	6	0.0010026	0.0042102	0.52383	175	3
TLE3	6	0.0010077	0.0042361	0.52383	176	4
TMEM147	6	0.0010119	0.0042502	0.52383	177	4
CUL3	6	0.0010283	0.0043169	0.52655	178	5
ZNF354C	6	0.0010292	0.0043205	0.52655	179	2
LIN7A	6	0.0010794	0.0045055	0.54604	180	2
DECR2	6	0.0011111	0.0046233	0.553996	181	3
NAALADL2	6	0.001115	0.0046391	0.553996	182	5
MUC21	6	0.0011169	0.0046473	0.553996	183	5
ZNF345	6	0.0011295	0.0046927	0.556366	184	2
HIST4H4	6	0.0011447	0.0047517	0.560316	185	2
CAND1	6	0.0011536	0.0047821	0.56087	186	4
SIPA1L3	6	0.0011727	0.004852	0.563645	187	4
TPD52	6	0.0011736	0.0048575	0.563645	188	5
AJUBA	6	0.0011971	0.0049505	0.569281	189	4
S100P	6	0.0012025	0.0049691	0.569281	190	4
PVRL2	6	0.0012096	0.0049904	0.569281	191	5
SLC35D2	6	0.0012153	0.0050127	0.569281	192	4
KCNN3	6	0.0012254	0.0050426	0.569281	193	4
EPO	6	0.0012299	0.0050626	0.569281	194	2
OR52N4	6	0.0012418	0.0051071	0.571338	195	5
SSTR3	6	0.0012578	0.0051629	0.572401	196	5
MICU2	6	0.0012744	0.0052255	0.572401	197	4
EPT1	6	0.0012744	0.0052255	0.572401	198	4
IRGQ	6	0.0012744	0.0052255	0.572401	199	4
KIAA1429	6	0.0012801	0.0052478	0.572401	200	3
WDR65	6	0.0012901	0.0052927	0.57443	201	5
WDR90	6	0.0012997	0.005329	0.575507	202	4
KPNA7	6	0.0013144	0.0053867	0.578867	203	4
FOSB	6	0.0013204	0.0054143	0.57899	204	5
RAD21	6	0.0013382	0.0054797	0.58312	205	4

ZNF385B	6	0.0013535	0.0055364	0.586297	206	4
HOOK3	6	0.0013793	0.0056336	0.589499	207	2
GJD3	6	0.0013797	0.0056349	0.589499	208	5
SLC2A11	6	0.0013926	0.0056776	0.589499	209	4
METTL12	6	0.0014064	0.0057302	0.589499	210	2
RSPH10B2	6	0.0014267	0.005801	0.589499	211	5
GSK3A	6	0.0014292	0.0058065	0.589499	212	5
FBXO9	6	0.0014304	0.0058115	0.589499	213	4
GYPC	6	0.0014306	0.0058124	0.589499	214	3
ANKRD53	6	0.0014362	0.0058319	0.589499	215	3
ACTN4	6	0.001438	0.0058369	0.589499	216	5
SIN3B	6	0.0014493	0.0058836	0.591481	217	5
CREB5	6	0.0014603	0.0059245	0.592856	218	4
CEP89	6	0.0014775	0.0059907	0.593286	219	4
ZNF324B	6	0.0014839	0.0060175	0.593286	220	4
AMBP	6	0.001484	0.006018	0.593286	221	3
TNFRSF12A	6	0.0015023	0.006091	0.593286	222	4
NUDT2	6	0.0015023	0.006091	0.593286	223	3
HIST1H3D	6	0.0015028	0.006092	0.593286	224	5
ANKHD1-EIF4E	5	0.0015162	0.0061405	0.595358	225	2
GP9	6	0.001547	0.0062513	0.603413	226	2
NUPR1L	6	0.0015811	0.0063856	0.613665	227	1
SCN2A	6	0.0015995	0.006456	0.617705	228	4
USP9X	6	0.0016305	0.0065676	0.623138	229	5
DMAP1	6	0.0016312	0.0065699	0.623138	230	4
ZNF45	6	0.0016466	0.0066375	0.626827	231	4
KCNN1	6	0.0016814	0.0067682	0.630939	232	3
RASA3	6	0.0016868	0.00679	0.630939	233	4
PCDHA9	2	0.0016969	0.0068272	0.630939	234	2
PSG3	6	0.0017005	0.0068408	0.630939	235	4
ZNF615	6	0.0017053	0.0068653	0.630939	236	3
TSC1	6	0.0017085	0.006874	0.630939	237	4
TEKT3	6	0.0017109	0.0068835	0.630939	238	4
FFAR2	6	0.0017308	0.0069547	0.632405	239	4
ICMT	6	0.0017315	0.0069575	0.632405	240	3
ASPHD1	6	0.0017567	0.0070573	0.635046	241	5
VN1R2	6	0.0017567	0.0070573	0.635046	242	5
ZNF514	6	0.0017695	0.0071077	0.635046	243	4
CSHL1	6	0.0017817	0.0071544	0.635046	244	1
hsa-mir-670	4	0.001782	0.0071549	0.635046	245	4
RAD9B	6	0.0017848	0.0071649	0.635046	246	3
DBR1	6	0.0017921	0.0071903	0.635046	247	5
ATP1A3	6	0.0018059	0.0072411	0.635137	248	3
DMKN	6	0.0018168	0.0072815	0.635137	249	4
AKT2	6	0.0018195	0.0072915	0.635137	250	3
IGFLR1	6	0.0018305	0.0073342	0.635137	251	5
CTPS2	6	0.0018318	0.0073369	0.635137	252	2
DLGAP2	6	0.0018591	0.0074395	0.641471	253	4
TMEM44	6	0.001882	0.007528	0.646546	254	1
FOXP2	6	0.0019036	0.0076119	0.647089	255	2
HYI	6	0.0019036	0.0076119	0.647089	256	4
C6orf15	6	0.0019071	0.0076233	0.647089	257	4

DFFB	6	0.001925	0.0076855	0.649839	258	4
CBL	6	0.0019367	0.0077299	0.650019	259	4
CNOT1	6	0.0019417	0.0077472	0.650019	260	4
NUCB1	6	0.0019674	0.007847	0.653259	261	4
KCNS2	6	0.0019674	0.007847	0.653259	262	3
ZNF302	6	0.0019823	0.0078933	0.653259	263	2
DGAT1	6	0.0019864	0.0079056	0.653259	264	4
JPH2	6	0.0020064	0.0079768	0.65666	265	2
KCNB1	6	0.0020324	0.0080803	0.662678	266	3
hsa-mir-1224	4	0.0020531	0.0081538	0.664013	267	4
DNPEP	6	0.0020545	0.0081575	0.664013	268	3
OR11L1	6	0.0020717	0.0082247	0.66527	269	2
TSTD2	6	0.0020826	0.0082691	0.66527	270	3
ZNF229	6	0.0020854	0.0082823	0.66527	271	4
DQX1	6	0.0020975	0.0083254	0.66527	272	2
BARD1	6	0.0020975	0.0083254	0.66527	273	3
NOA1	6	0.0021144	0.0083885	0.666877	274	4
MGEA5	6	0.0021194	0.0084067	0.666877	275	5
ZNF812	6	0.0021838	0.0086667	0.675858	276	5
EPHA7	6	0.0021866	0.0086758	0.675858	277	5
CNOT11	6	0.0021901	0.0086876	0.675858	278	5
TCF19	6	0.0021919	0.0086935	0.675858	279	4
RPS25	6	0.0022022	0.0087384	0.675858	280	3
hsa-mir-3194	4	0.0022198	0.0087947	0.675858	281	2
GNG8	6	0.0022223	0.0088042	0.675858	282	3
GPR19	6	0.0022319	0.0088424	0.675858	283	5
SFRP5	6	0.002233	0.0088451	0.675858	284	2
OR8U8	5	0.0022539	0.008915	0.675858	285	3
TPTE2	6	0.0022639	0.0089458	0.675858	286	3
GNG7	6	0.0022786	0.0089944	0.675858	287	5
FEM1C	6	0.0022831	0.0090062	0.675858	288	1
MYO7A	6	0.0022933	0.0090475	0.675858	289	3
TRMT2A	6	0.0022941	0.0090516	0.675858	290	5
hsa-mir-381	4	0.0022943	0.009052	0.675858	291	4
SCHIP1	4	0.0023098	0.009111	0.675858	292	3
SDR42E1	6	0.0023154	0.0091324	0.675858	293	2
CCDC92	6	0.0023154	0.0091324	0.675858	294	3
APOBR	6	0.0023262	0.0091696	0.675858	295	5
FAM151A	6	0.0023314	0.00919	0.675858	296	4
ZNF714	6	0.0023396	0.0092222	0.675858	297	4
TGFBR2	6	0.0023476	0.0092522	0.675858	298	4
NEURL	6	0.0023583	0.0092935	0.675858	299	2
hsa-mir-217	4	0.0023585	0.0092944	0.675858	300	2
HINFP	6	0.0023793	0.009372	0.677285	301	3
MFSD6	6	0.0024114	0.0094877	0.677285	302	3
TMEM200A	6	0.0024184	0.0095118	0.677285	303	5
HUWE1	6	0.0024268	0.0095431	0.677285	304	4
DCAF15	6	0.0024284	0.009549	0.677285	305	4
RSC1A1	6	0.002435	0.0095758	0.677285	306	4
IFFO2	6	0.0024406	0.0095953	0.677285	307	5
CXCR5	6	0.0024461	0.0096166	0.677285	308	4
HPN	6	0.00245	0.0096298	0.677285	309	4

Supplementary table 3: Function of top 158 genes (FDR<0.5) according to STRING DB		
**input rank**	**mapped gene**	**annotation**
1	KLF1	Kruppel-like factor 1 (erythroid); Transcription regulator of erythrocyte development that probably serves as a general switch factor during erythropoiesis. Is a dual regulator of fetal-to-adult [...]
2	BAX	BCL2-associated X protein; Accelerates programmed cell death by binding to, and antagonizing the apoptosis repressor BCL2 or its adenovirus homolog E1B 19k protein. Under stress conditions, under [...]
3	BAP1	BRCA1 associated protein-1 (ubiquitin carboxy-terminal hydrolase); Deubiquitinating enzyme that plays a key role in chromatin by mediating deubiquitination of histone H2A and HCFC1. Catalytic com [...]
4	EIF2AK1	Eukaryotic translation initiation factor 2-alpha kinase 1; Inhibits protein synthesis at the translation initiation level, in response to various stress conditions, including oxidative stress, he [...]
5	MED24	Mediator complex subunit 24; Component of the Mediator complex, a coactivator involved in the regulated transcription of nearly all RNA polymerase II-dependent genes. Mediator functions as a brid [...]
6	BCL2L11	BCL2-like 11 (apoptosis facilitator); Induces apoptosis and anoikis. Isoform BimL is more potent than isoform BimEL. Isoform Bim-alpha1, isoform Bim-alpha2 and isoform Bim-alpha3 induce apoptosis [...]
7	SPRED2	Sprouty-related, EVH1 domain containing 2; Tyrosine kinase substrate that inhibits growth-factor- mediated activation of MAP kinase
8	UBE2M	Ubiquitin-conjugating enzyme E2M; Accepts the ubiquitin-like protein NEDD8 from the UBA3- NAE1 E1 complex and catalyzes its covalent attachment to other proteins. The specific interaction with th [...]
9	SMARCA5	SWI/SNF related, matrix associated, actin dependent regulator of chromatin, subfamily a, member 5; Helicase that possesses intrinsic ATP-dependent nucleosome-remodeling activity. Complexes contai [...]
10	DMRTC2	DMRT-like family C2; May be involved in sexual development
11	TGFBR1	Transforming growth factor, beta receptor 1; Transmembrane serine/threonine kinase forming with the TGF-beta type II serine/threonine kinase receptor, TGFBR2, the non-promiscuous receptor for the [...]
12	NF1	Neurofibromin 1; Stimulates the GTPase activity of Ras. NF1 shows greater affinity for Ras GAP, but lower specific activity. May be a regulator of Ras activity
13	APAF1	Apoptotic peptidase activating factor 1; Oligomeric Apaf-1 mediates the cytochrome c-dependent autocatalytic activation of pro-caspase-9 (Apaf-3), leading to the activation of caspase-3 and apopt [...]
14	SLC30A1	Solute carrier family 30 (zinc transporter), member 1; May be involved in zinc transport out of the cell
15	miRNA 142	miRNA molecule

16	TMEM163	Transmembrane protein 163; May bind zinc and other divalent cations and recruit them to vesicular organelles
17	FBXO17	F-box protein 17; Substrate-recognition component of the SCF (SKP1-CUL1-F- box protein)-type E3 ubiquitin ligase complex. Able to recognize and bind denatured glycoproteins, which are modified wi [...]
18	TARBP2	TAR (HIV-1) RNA binding protein 2; Required for formation of the RNA induced silencing complex (RISC). Component of the RISC loading complex (RLC), also known as the micro-RNA (miRNA) loading com [...]
19	BLVRB	Biliverdin reductase B (flavin reductase (NADPH)); Broad specificity oxidoreductase that catalyzes the NADPH-dependent reduction of a variety of flavins, such as riboflavin, FAD or FMN, biliverdi [...]
20	WDR87	WD repeat domain 87
21	SNX9	Sorting nexin 9; Involved in endocytosis and intracellular vesicle trafficking, both during interphase and at the end of mitosis. Required for efficient progress through mitosis and cytokinesis. [...]
22	PMAIP1	Phorbol-12-myristate-13-acetate-induced protein 1; Promotes activation of caspases and apoptosis. Promotes mitochondrial membrane changes and efflux of apoptogenic proteins from the mitochondria. [...]
23	KPTN	Kaptin (actin binding protein); Necessary for normal neuromorphogenesis. May be involved in actin dynamics. May play a role in producing the sensory apparatus in hair cells. May play a role in ac [...]
24	RREB1	Ras responsive element binding protein 1
25	HBZ	Hemoglobin, zeta; The zeta chain is an alpha-type chain of mammalian embryonic hemoglobin
26	TSC2	Tuberous sclerosis 2; In complex with TSC1, this tumor suppressor inhibits the nutrient-mediated or growth factor-stimulated phosphorylation of S6K1 and EIF4EBP1 by negatively regulating mTORC1 s [...]
27	NPRL3	Nitrogen permease regulator-like 3 (S. cerevisiae); As a component of the GATOR1 complex, inhibitor of the amino acid-sensing branch of the TORC1 pathway. The GATOR1 complex strongly increases GT [...]
28	DEPDC5	DEP domain containing 5; As a component of the GATOR1 complex, inhibitor of the amino acid-sensing branch of the TORC1 pathway. The GATOR1 complex strongly increases GTP hydrolysis by RRAGA and R [...]
29	SMARCD1	SWI/SNF related, matrix associated, actin dependent regulator of chromatin, subfamily d, member 1; Involved in chromatin remodeling. Belongs to the neural progenitors-specific chromatin remodelin [...]
30	ORC2	Origin recognition complex, subunit 2; Component of the origin recognition complex (ORC) that binds origins of replication. DNA-binding is ATP-dependent. The specific DNA sequences that define or [...]
31	ZNF341	Zinc finger protein 341; May be involved in transcriptional regulation
32	CXCL17	Chemokine (C-X-C motif) ligand 17; Plays a role in angiogenesis and possibly in the development of tumors. May be a housekeeping chemokine regulating recruitment of nonactivated blood monocytes a [...]

33	PCGF1	Polycomb group ring finger 1; Component of the Polycomb group (PcG) multiprotein BCOR complex, a complex required to maintain the transcriptionally repressive state of some genes, such as BCL6 an [...]
34	RALGAPA2	Ral GTPase activating protein, alpha subunit 2 (catalytic); Catalytic subunit of the heterodimeric RalGAP2 complex which acts as a GTPase activator for the Ras-like small GTPases RALA and RALB
35	ERCC2	Excision repair cross-complementing rodent repair deficiency, complementation group 2; ATP-dependent 5'-3' DNA helicase, component of the core- TFIIH basal transcription factor. Involved in nucle [...]
36	YIF1B	Yip1 interacting factor homolog B (S. cerevisiae)
37	WDR33	WD repeat domain 33; Essential for both cleavage and polyadenylation of pre-mRNA 3' ends
38	USP22	Ubiquitin specific peptidase 22; Histone deubiquitinating component of the transcription regulatory histone acetylation (HAT) complex SAGA. Catalyzes the deubiquitination of both histones H2A and [...]
39	miRNA 6888	miRNA molecule
40	PTEN	Phosphatase and tensin homolog; Tumor suppressor. Acts as a dual-specificity protein phosphatase, dephosphorylating tyrosine-, serine- and threonine-phosphorylated proteins. Also acts as a lipid [...]
41	TXNDC2	Thioredoxin domain containing 2 (spermatozoa); Probably plays a regulatory role in sperm development. May participate in regulation of fibrous sheath (FS) assembly by supporting the formation of [...]
42	PPP5D1	PPP5 tetratricopeptide repeat domain containing 1
43	RGS11	Regulator of G-protein signaling 11; Inhibits signal transduction by increasing the GTPase activity of G protein alpha subunits thereby driving them into their inactive GDP-bound form
44	MDN1	MDN1, midasin homolog (yeast); Nuclear chaperone required for maturation and nuclear export of pre-60S ribosome subunits
45	CPSF1	Cleavage and polyadenylation specific factor 1, 160kDa; Component of the cleavage and polyadenylation specificity factor (CPSF) complex that plays a key role in pre- mRNA 3'-end formation, recogn [...]
46	TAS2R43	Taste receptor, type 2, member 43; Gustducin-coupled receptor immplicated in the perception of bitter compounds in the oral cavity and the gastrointestinal tract. Signals through PLCB2 and the ca [...]
47	CASP3	Caspase 3, apoptosis-related cysteine peptidase; Involved in the activation cascade of caspases responsible for apoptosis execution. At the onset of apoptosis it proteolytically cleaves poly(ADP- [...]
48	PLGLB2	Plasminogen-like B2; May bind noncovalently to lysine binding sites present in the kringle structures of plasminogen. This may interfere with the binding of fibrin or alpha-2-antiplasmin to plasm [...]
49	FEZF1	FEZ family zinc finger 1; Transcription repressor. Involved in the axonal projection and proper termination of olfactory sensory neurons (OSN). Plays a role in rostro-caudal patterning of the die [...]

50	SSX7	Synovial sarcoma, X breakpoint 7; Could act as a modulator of transcription
51	UBE2Q1	Ubiquitin-conjugating enzyme E2Q family member 1; Catalyzes the covalent attachment of ubiquitin to other proteins (Potential). May be involved in hormonal homeostasis in females. Involved in reg [...]
52	RPS4Y2	Ribosomal protein S4, Y-linked 2
53	LRRCC1	Leucine rich repeat and coiled-coil centrosomal protein 1; Required for the organization of the mitotic spindle. Maintains the structural integrity of centrosomes during mitosis
54	ERCC1	Excision repair cross-complementing rodent repair deficiency, complementation group 1 (includes overlapping antisense sequence); Isoform 1: Non-catalytic component of a structure- specific DNA re [...]
55	LRRK1	Leucine-rich repeat kinase 1
56	MED12	Mediator complex subunit 12; Component of the Mediator complex, a coactivator involved in the regulated transcription of nearly all RNA polymerase II-dependent genes. Mediator functions as a brid [...]
57	CDCA7	Cell division cycle associated 7; Participates in MYC-mediated cell transformation and apoptosis; induces anchorage-independent growth and clonogenicity in lymphoblastoid cells. Insufficient to i [...]
58	CCZ1B	CCZ1 vacuolar protein trafficking and biogenesis associated homolog B (S. cerevisiae)
59	PSG5	Pregnancy specific beta-1-glycoprotein 5
60	ESM1	Endothelial cell-specific molecule 1; Involved in angiogenesis; promotes angiogenic sprouting. May have potent implications in lung endothelial cell-leukocyte interactions
61	IRX4	Iroquois homeobox 4; Likely to be an important mediator of ventricular differentiation during cardiac development
62	USF2	Upstream transcription factor 2, c-fos interacting; Transcription factor that binds to a symmetrical DNA sequence (E-boxes) (5'-CACGTG-3') that is found in a variety of viral and cellular promote [...]
63	PRKDC	Protein kinase, DNA-activated, catalytic polypeptide; Serine/threonine-protein kinase that acts as a molecular sensor for DNA damage. Involved in DNA non-homologous end joining (NHEJ) required fo [...]
64	GSTCD	Glutathione S-transferase, C-terminal domain containing
65	TCEB1	Transcription elongation factor B (SIII), polypeptide 1 (15kDa, elongin C); SIII, also known as elongin, is a general transcription elongation factor that increases the RNA polymerase II transcri [...]
66	ZNF235	Zinc finger protein 235; May be involved in transcriptional regulation

67	ZNF382	Zinc finger protein 382; Functions as a sequence-specific transcriptional repressor
68	CYP2A13	Cytochrome P450, family 2, subfamily A, polypeptide 13; Exhibits a coumarin 7-hydroxylase activity. Active in the metabolic activation of hexamethylphosphoramide, N,N- dimethylaniline, 2'-methoxy [...]
69	FAM159A	Family with sequence similarity 159, member A
70	DPY19L3	Dpy-19-like 3 (C. elegans); Probable C-mannosyltransferase that mediates C-mannosylation of tryptophan residues on target proteins
71	BBC3	BCL2 binding component 3; Essential mediator of p53/TP53-dependent and p53/TP53- independent apoptosis. Isoform 3 fails to show any growth- inhibitory or apoptotic activity
72	RBM44	RNA binding motif protein 44; Component of intercellular bridges during meiosis. Intercellular bridges are evolutionarily conserved structures that connect differentiating germ cells. Not require [...]
73	NME4	NME/NM23 nucleoside diphosphate kinase 4; Major role in the synthesis of nucleoside triphosphates other than ATP. The ATP gamma phosphate is transferred to the NDP beta phosphate via a ping-pong [...]
74	FAM59A	Family with sequence similarity 59, member A; Isoform 1: Acts as an adapter protein that plays a role in intracellular signaling cascades triggered either by the cell surface activated epidermal [...]
75	TACO1	Translational activator of mitochondrially encoded cytochrome c oxidase I; Acts as a translational activator of mitochondrially- encoded cytochrome c oxidase 1
76	LIPG	Lipase, endothelial; Has phospholipase and triglyceride lipase activities. Hydrolyzes high density lipoproteins (HDL) more efficiently than other lipoproteins. Binds heparin
77	C11orf35	Chromosome 11 open reading frame 35
78	CC2D1A	Coiled-coil and C2 domain containing 1A; Transcription factor that binds specifically to the DRE (dual repressor element) and represses HTR1A gene transcription in neuronal cells. The combination [...]
79	C1QTNF3	C1q and tumor necrosis factor related protein 3
80	PF4	Platelet factor 4; Released during platelet aggregation. Neutralizes the anticoagulant effect of heparin because it binds more strongly to heparin than to the chondroitin-4-sulfate chains of the [...]
81	KCTD5	Potassium channel tetramerisation domain containing 5; Its interaction with CUL3 suggests that it may act as a substrate adapter in some E3 ligase complex. Does not affect the function of Kv chan [...]
82	HSPA1B	Heat shock 70kDa protein 1B
83	RBAK	RB-associated KRAB zinc finger; May repress E2F-dependent transcription. May promote AR- dependent transcription

84	FSTL3	Follistatin-like 3 (secreted glycoprotein); Isoform 1 or the secreted form is a binding and antagonizing protein for members of the TGF-beta family, such us activin, BMP2 and MSTN. Inhibits activ [...]
85	FXYD3	FXYD domain containing ion transport regulator 3; Induces a hyperpolarization-activated chloride current when expressed in Xenopus oocytes. May be a modulator capable of activating endogenous ooc [...]
86	MSMO1	Methylsterol monooxygenase 1; Catalyzes the first step in the removal of the two C-4 methyl groups of 4,4-dimethylzymosterol
87	SMCHD1	Structural maintenance of chromosomes flexible hinge domain containing 1; Required for maintenance of X inactivation in females and hypermethylation of CpG islands associated with inactive X. Inv [...]
88	ZNF155	Zinc finger protein 155; May be involved in transcriptional regulation
89	CYP27A1	Cytochrome P450, family 27, subfamily A, polypeptide 1; Catalyzes the first step in the oxidation of the side chain of sterol intermediates; the 27-hydroxylation of 5-beta- cholestane-3-alpha,7-a [...]
90	PRSS1	Protease, serine, 1 (trypsin 1); Has activity against the synthetic substrates Boc-Phe- Ser-Arg-Mec, Boc-Leu-Thr-Arg-Mec, Boc-Gln-Ala-Arg-Mec and Boc-Val- Pro-Arg-Mec. The single-chain form is mo [...]
91	MED4	Mediator complex subunit 4; Component of the Mediator complex, a coactivator involved in the regulated transcription of nearly all RNA polymerase II-dependent genes. Mediator functions as a bridg [...]
92	LZTR1	Leucine-zipper-like transcription regulator 1; Probable transcriptional regulator that may play a crucial role in embryogenesis
93	miRNA 6088	miRNA molecule
94	SAMD4B	Sterile alpha motif domain containing 4B; Has transcriptional repressor activity. Overexpression inhibits the transcriptional activities of AP-1, p53/TP53 and CDKN1A
95	ADIPOR2	Adiponectin receptor 2; Receptor for ADIPOQ, an essential hormone secreted by adipocytes that regulates glucose and lipid metabolism (PubMed:12802337, PubMed:25855295). Required for normal body f [...]
96	KLC3	Kinesin light chain 3; Kinesin is a microtubule-associated force-producing protein that may play a role in organelle transport
97	TMEM67	Transmembrane protein 67; Required for ciliary structure and function. Part of the tectonic-like complex which is required for tissue-specific ciliogenesis and may regulate ciliary membrane compo [...]
98	RUNX1T1	Runt-related transcription factor 1; translocated to, 1 (cyclin D-related); Transcriptional corepressor which facilitates transcriptional repression via its association with DNA-binding transcrip [...]
99	ZNF585A	Zinc finger protein 585A; May be involved in transcriptional regulation
100	MAOA	Monoamine oxidase A; Catalyzes the oxidative deamination of biogenic and xenobiotic amines and has important functions in the metabolism of neuroactive and vasoactive amines in the central nervou [...]

101	DYSF	Dysferlin, limb girdle muscular dystrophy 2B (autosomal recessive); Key calcium ion sensor involved in the Ca(2+)-triggered synaptic vesicle-plasma membrane fusion. Plays a role in the sarcolemma [...]
102	HKR1	HKR1, GLI-Kruppel zinc finger family member; May be involved in transcriptional regulation
103	DOK4	Docking protein 4; DOK proteins are enzymatically inert adaptor or scaffolding proteins. They provide a docking platform for the assembly of multimolecular signaling complexes. DOK4 functions in [...]
104	CNIH4	Cornichon homolog 4 (Drosophila); Involved in G protein-coupled receptors (GPCRs) trafficking from the endoplasmic reticulum to the cell surface; it promotes the exit of GPCRs from the early secr [...]
105	MED19	Mediator complex subunit 19; Component of the Mediator complex, a coactivator involved in the regulated transcription of nearly all RNA polymerase II-dependent genes. Mediator functions as a brid [...]
106	NEK9	NIMA-related kinase 9; Pleiotropic regulator of mitotic progression, participating in the control of spindle dynamics and chromosome separation. Phosphorylates different histones, myelin basic pr [...]
107	ADAM30	ADAM metallopeptidase domain 30; May be involved in spermatogenesis and fertilization
108	TGFB2	Transforming growth factor, beta 2
109	HBG1	Hemoglobin, gamma A
110	MBD1	methyl-CpG binding domain protein 1; Transcriptional repressor that binds CpG islands in promoters where the DNA is methylated at position 5 of cytosine within CpG dinucleotides. Binding is aboli [...]
111	FAM129B	Family with sequence similarity 129, member B; May play a role in apoptosis suppression. May promote melanoma cell invasion in vitro
112	FAM217B	Family with sequence similarity 217, member B
113	DHX29	DEAH (Asp-Glu-Ala-His) box polypeptide 29; ATP-binding RNA helicase involved in translation initiation. Part of the 43S preinitiation complex that is required for efficient initiation on mammalia [...]
114	FOXR1	Forkhead box R1
115	VPS13C	Vacuolar protein sorting 13 homolog C (S. cerevisiae)
116	HBG2	Hemoglobin, gamma G; Gamma chains make up the fetal hemoglobin F, in combination with alpha chains
117	MED16	Mediator complex subunit 16; Component of the Mediator complex, a coactivator involved in the regulated transcription of nearly all RNA polymerase II-dependent genes. Mediator functions as a brid [...]

118	CPSF2	Cleavage and polyadenylation specific factor 2, 100kDa; Component of the cleavage and polyadenylation specificity factor (CPSF) complex that play a key role in pre-mRNA 3'-end formation, recogniz [...]
119	MED23	Mediator complex subunit 23; Required for transcriptional activation subsequent to the assembly of the pre-initiation complex (By similarity). Component of the Mediator complex, a coactivator inv [...]
120	MLF2	Myeloid leukemia factor 2
121	FBXL19	F-box and leucine-rich repeat protein 19; Substrate-recognition component of the SCF (SKP1-CUL1-F- box protein)-type E3 ubiquitin ligase complex
122	UNC119B	Unc-119 homolog B (C. elegans); Myristoyl-binding protein that acts as a cargo adapter: specifically binds the myristoyl moiety of a subset of N- terminally myristoylated proteins and is required [...]
123	AIDA	Axin interactor, dorsalization associated; Acts as a ventralizing factor during embryogenesis. Inhibits axin-mediated JNK activation by binding axin and disrupting axin homodimerization. This in [...]
124	VPS13D	Vacuolar protein sorting 13 homolog D (S. cerevisiae)
125	NUMA1	Nuclear mitotic apparatus protein 1; Highly abundant component of the nuclear matrix where it may serve a non-mitotic structural role, occupies the majority if the nuclear volume (PubMed:10075938 [...]
126	DHRS9	Dehydrogenase/reductase (SDR family) member 9; 3-alpha-hydroxysteroid dehydrogenase that converts 3- alpha-tetrahydroprogesterone (allopregnanolone) to dihydroxyprogesterone and 3-alpha-androstan [...]
127	SEMA6B	Sema domain, transmembrane domain (TM), and cytoplasmic domain, (semaphorin) 6B; May play a role in both peripheral and central nervous system development
128	CABIN1	Calcineurin binding protein 1; May be required for replication-independent chromatin assembly. May serve as a negative regulator of T-cell receptor (TCR) signaling via inhibition of calcineurin. [...]
129	miRNA 4742	miRNA molecule
130	CLEC2D	C-type lectin domain family 2, member D
131	CSTF3	Cleavage stimulation factor, 3' pre-RNA, subunit 3, 77kDa; One of the multiple factors required for polyadenylation and 3'-end cleavage of mammalian pre-mRNAs
132	ARHGAP35	Rho GTPase activating protein 35; Represses transcription of the glucocorticoid receptor by binding to the cis-acting regulatory sequence 5'-GAGAAAAGAAACTGGAGAAACTC-3'. May participate in the re [...]
133	FAM108B1	Family with sequence similarity 108, member B1
134	SORCS2	Sortilin-related VPS10 domain containing receptor 2

135	TTC9B	Tetratricopeptide repeat domain 9B
136	CRYBB3	Crystallin, beta B3; Crystallins are the dominant structural components of the vertebrate eye lens
137	RCE1	RCE1 homolog, prenyl protein protease (S. cerevisiae); Proteolytically removes the C-terminal three residues of farnesylated and geranylated proteins. Seems to be able to process K-Ras, N-Ras, H- [...]
138	CD2	CD2 molecule; CD2 interacts with lymphocyte function-associated antigen (LFA-3) and CD48/BCM1 to mediate adhesion between T-cells and other cell types. CD2 is implicated in the triggering of T- c [...]
139	C19orf40	Chromosome 19 open reading frame 40; Plays a role in DNA repair through recruitment of the FA core complex to damaged DNA. Regulates FANCD2 monoubiquitination upon DNA damage. Induces chromosomal [...]
140	ZC3HC1	Zinc finger, C3HC-type containing 1; Essential component of an SCF-type E3 ligase complex, SCF(NIPA), a complex that controls mitotic entry by mediating ubiquitination and subsequent degradation [...]
141	RNF7	Ring finger protein 7; Probable component of the SCF (SKP1-CUL1-F-box protein) E3 ubiquitin ligase complex which mediates the ubiquitination and subsequent proteasomal degradation of target prote [...]
142	GTF3A	General transcription factor IIIA; Interacts with the internal control region (ICR) of approximately 50 bases within the 5S RNA genes, is required for correct transcription of these genes by RNA [...]
143	MFSD9	Major facilitator superfamily domain containing 9
144	ZNF99	Zinc finger protein 99
145	BIN2	Bridging integrator 2; Promotes cell motility and migration, probably via its interaction with the cell membrane and with podosome proteins that mediate interaction with the cytoskeleton. Modulat [...]
146	CMTM8	CKLF-like MARVEL transmembrane domain containing 8
147	CALR3	Calreticulin 3; During spermatogenesis, may act as a lectin-independent chaperone for specific client proteins such as ADAM3. Required for sperm fertility (By similarity). CALR3 capacity for calc [...]
148	TMTC3	Transmembrane and tetratricopeptide repeat containing 3
149	IQCG	IQ motif containing G
150	miRNA 4799	miRNA molecule
151	GABPB2	GA binding protein transcription factor, beta subunit 2; May function as transcription factor capable of interacting with purine rich repeats (GA repeats)

152	LPPR1	Lipid phosphate phosphatase-related protein type 1
153	CALM3	Calmodulin 3 (phosphorylase kinase, delta)
154	CILP	Cartilage intermediate layer protein, nucleotide pyrophosphohydrolase; Probably plays a role in cartilage scaffolding. May act by antagonizing TGF-beta1 (TGFB1) and IGF1 functions. Has the abilit [...]
155	ANAPC15	Anaphase promoting complex subunit 15; Component of the anaphase promoting complex/cyclosome (APC/C), a cell cycle-regulated E3 ubiquitin ligase that controls progression through mitosis and the [...]
156	IAH1	Isoamyl acetate-hydrolyzing esterase 1 homolog (S. cerevisiae); Probable lipase
157	miRNA 548f-3	miRNA molecule
158	SHPK	Sedoheptulokinase; Acts as a modulator of macrophage activation through control of glucose metab
